# Seasonal Dynamics of the Gut Microbiome in Urban Feral Pigeons Are Associated With Environmental Conditions, Not With Diet Shifts

**DOI:** 10.1002/ece3.73682

**Published:** 2026-05-13

**Authors:** Kangqing Zhang, Henri Zomer, Gerrit Potkamp, Joana Falcao Salles, B. Irene Tieleman, Maurine W. Dietz

**Affiliations:** ^1^ Groningen Institute for Evolutionary Life Sciences University of Groningen Groningen the Netherlands; ^2^ Aestas Ecologie Haulerwijk the Netherlands

**Keywords:** avian microbiome, diet, feral pigeon, seasonal variation, urbanization

## Abstract

Gut microbiomes play a crucial role in host physiology and seasonal adaptation. While seasonal shifts in avian gut microbiota are often attributed to seasonal diet variation, environmental factors may be equally or more important, particularly in urban ecosystems. This study aimed to determine whether seasonal variation in the gut microbiome of free‐living feral pigeons (*Columba livia f. domestica*) inhabiting urban environments is associated with seasonal changes in diet and environmental conditions. We captured feral pigeons at three locations in Groningen, the Netherlands, during winter (January–February 2019) and summer (July–August 2019). Cloacal swabs and fecal samples were collected to assess gut microbiota via 16S rRNA sequencing and diet via DNA metabarcoding, respectively. Microbial diversity and composition showed significant seasonal variation and location effects. At Vismarkt, one of the three urban sampling sites within the city of Groningen, Firmicutes were more abundant in summer than in winter, while Actinobacteria were more abundant in winter. Dominant genera also varied seasonally, with *Lactobacillus* more abundant in summer. In contrast, the diet composition was dominated by *Poaceae* (grasses), *Fabaceae* (legumes), and *Asteraceae* (daisies) across all seasons and locations, with no detectable differences between locations or seasons. Distance‐based redundancy analysis indicated that temperature was significantly associated with microbiome composition, whereas diet as measured here showed no detectable association. This suggests that seasonal microbiome variation in urban feral pigeons may be related to seasonal environmental conditions even without detectable dietary shifts, consistent with the idea that seasonal environmental conditions can contribute to microbiome seasonality in birds.

## Graphical abstract

Using feral pigeons in urban environments, we show that microbiome diversity and composition vary seasonally despite stable diet composition.

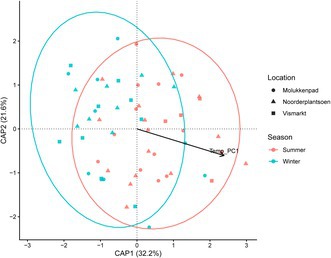

## 1 Introduction

The gut microbiome, comprising bacteria, archaea, viruses, fungi, and other microorganisms inhabiting the gastrointestinal tract, has emerged as a critical factor in the physiological and behavioral adaptations of animals, influencing diverse life‐history traits such as immunity, growth, and seasonal phenotypic shifts (van Veelen et al. 2020; van Veelen et al. 2018; Zhu et al. 2021; van Veelen et al. 2022). In birds, gut microbiome composition is especially dynamic, adapting in response to changes in food availability and environmental conditions (Loo et al. 2019; van Veelen et al. 2023). Dietary choices have a significant impact on the microbiome, often surpassing the influence of host genetics (Song et al. 2020). Research on the avian gut microbiome has demonstrated that seasonal diet shifts often lead to corresponding changes in microbial diversity and composition (Teyssier et al. 2020; Schmiedova et al. 2022; Xiao et al. 2021). Generally, dietary changes impact the gut microbiome directly, where microorganisms in consumed items influence the microbiome, or indirectly, where shifts in nutrient availability affect gut microbiome competition and community structure (Wolff et al. 2017). In non‐passerine birds, for example, diets based on mixed corn‐soybean foster a microbial community featuring Clostridia and Bacteroidia. Starch‐heavy diets, on the other hand, boost *Lactobacillus* levels, whereas high‐fiber diets increase *Clostridium* levels. Conversely, carnivorous diets elevate Fusobacteria and Proteobacteria levels (Xiao et al. 2021).

Seasonal changes in the gut microbiome are mainly attributed to fluctuations in dietary resources, which can be influenced by environmental factors like weather and vegetation that also change seasonally (Lewis et al. 2017; Lewis et al. 2016). However, abiotic environmental factors, such as temperature, precipitation, and day length, may also directly drive variation in gut microbiome diversity and function (Chevalier et al. 2015; Bailey et al. 2010). For example, the gut microbiome community in captive homing pigeons (*Columba livia f. domestica*), kept in outdoor aviaries, undergoes seasonal changes, despite a constant diet (Dietz et al. 2022). Other studies have shown that heat stress induces significant alterations in the gut microbiome of poultry, with a decrease in Firmicutes and an increase in Bacteroidetes under high‐temperature conditions (Zhu et al. 2019). Light exposure also plays a role; in Siberian hamsters ( *Phodopus sungorus* ), shorter photoperiods are associated with changes in gut bacterial composition, including shifts in the relative abundances of key taxa, such as Proteobacteria and Firmicutes (Bailey et al. 2010; Shor et al. 2020).

Host‐microbiome‐environment interactions are particularly significant in the context of urban wildlife, where birds are exposed to unique environmental pressures, including human‐modified food sources and urban microclimates. Although animals experience the same seasonal variation in day length when living in cities and natural areas, cities are in general warmer (urban heating effect, Rizwan et al. 2008) and provide more constant microclimates and food resources to their inhabitants throughout the year (Luniak 2004; Maciusik et al. 2009). In addition, habitat disturbance and exposure to humans can significantly influence the microbes found in avian intestines. For example, in White‐crowned Sparrows ( *Zonotrichia leucophrys* ), the urban gut microbiome is more diverse than the rural gut microbiome, a finding hypothesized to be due to heterogeneous land cover and altered food resources in cities (Phillips et al. 2018). Similarly, house sparrows ( *Passer domesticus* ) in cities were more enriched with Proteobacteria than rural birds (Gadau et al. 2019). Overall, the impact of human activities on host habitats can disrupt the relationship between host‐gut microbiome communities.

Despite recent advances, it remains unclear how urban birds respond to seasonal changes at the level of the gut microbiome. In particular, little is known about whether seasonal shifts in urban bird microbiomes are driven by diet season changes, by abiotic environmental factors such as temperature and daylength, or by a combination of both (Schmiedova et al. 2022; Góngora et al. 2021). To address this, we investigated seasonal variation in the gut microbiome and diet of feral pigeons, a resident urban species that experiences full annual cycles under city conditions (Obukhova 2007; Corbel et al. 2016). Specifically, we investigated whether seasonal microbiome shifts are associated with diet, seasonal variations in environmental conditions, or their interaction. We captured pigeons in winter and summer at three locations in the city of Groningen, the Netherlands, and collected cloacal swabs to determine the gut microbiome via 16S rRNA gene sequencing, as well as fecal samples to assess diet via metabarcoding.

## 2 Material and Methods

### 2.1 Site Description and Sample Collection

We captured feral pigeons in winter (January and February) and summer (June to August) of 2019 at Vismarkt (53°13′02″ N, 6°33′53″ E), Noorderplantsoen (53°13′16″ N, 6°33′15″ E), and Molukkenpad (53°14′08″ N, 6°34′40″ E) in the city of Groningen, The Netherlands.

Vismarkt is a large, paved square located in the city center (ca 7000 m^2^), used for markets 5 days a week, and has a relatively small pigeon population of around 30 individuals. Noorderplantsoen is a large city park featuring extensive vegetation, including large trees, lawns, and four ponds, covering roughly 200,000 m^2^ and supporting an estimated 100 pigeons. Molukkenpad is a suburban park containing trees, grass, flower beds, and two large ponds (ca 65,000 m^2^), and a pigeon population of approximately 30 individuals. Compared with Vismarkt, both parks contain substantially more vegetation and water bodies. Capture locations were located between 0.8 and 2.3 km apart. Birds were caught individually using a hand‐held noose and bait to attract them. Re‐sightings of previously captured and banded feral pigeons, suggest that feral pigeons are highly site and group‐faithful in Groningen, as birds captured at one site are rarely seen at other capture sites. We were only able to recapture 7 of the 56 pigeons in winter and summer.

After capture, the pigeons were banded with two rings, each with a unique code, an aluminum ring and a color ring, allowing for identification in the following season (pursuant to ringing permit no. 951, issued on 1 March 1995 by the Minister of LNV to the Dutch Ringing Centre/Vogeltrekstation of the Netherlands Institute for Ecology). We scored body mass (325.94 ± 4.56 g), head bill (53.78 ± 0.16 mm), and wing length (234.49 ± 0.86 mm). We took a cloacal swab for gut microbiome analysis by inserting a sterile viscous swab (Cultiplast, LP Italiana SpA, Italy; catalog no. 112598) into the cloaca and gently rotating it for 10 s within the cloacal lumen. To minimize external contamination, care was taken to avoid contact with feathers or external skin surfaces, and swabs were discarded and replaced if accidental contact occurred. All tools and working surfaces were disinfected with 70% ethanol, and gloves were worn and regularly disinfected during sampling. The swab was placed in the sterile vial and stored on ice. Additionally, we collected a negative control swab every capture day at a capture location and stored it in a sterile vial. Upon arrival at the lab, 25 μL of sterile PBS solution was added to the swabs before storing them at −80°C (*n* = 73, including 63 cloacal swabs and 10 negative controls). Lastly, we collected feces to assess diet by placing the feral pigeons in a closed box for 5 min. The bottom of the box was lined with a UV‐sterilized backing paper sheet, which was replaced by a new sheet for each individual. Using a disinfected spoon, the feces were collected in a sterilized vial (*n* = 71, comprising 63 feces samples and 8 field negative controls). Upon arrival at the lab, the fecal samples were stored at −80°C. The experimental procedures used in this study, taking host traits measures, taking a cloacal swab, collecting a dropping, and plucking one breast feather, did not exceed the legal discomfort threshold for animal experiments, and thus this study was not considered an animal experiment under Dutch law (Animal Welfare Body of the University of Groningen).

We obtained environmental indices, including daily mean, minimum, and maximum temperature (in 0.1°C), precipitation duration (in 0.1 h), and daily precipitation amount (in 0.1 mm) from weather station 280 of the Royal Netherlands Meteorological Institute (KNMI; located ca. 10.5–12 km south of the capture locations at Eelde; N 53° 7.674′, E 6° 35.152′), for the sample collection dates. Data are available at: https://www.knmi.nl/nederland‐nu/klimatologie/daggegevens. Sunrise and sunset times were retrieved from https://www.sunrise‐and‐sunset.com/nl/sun/nederland/Groningen, and day length (hours) was calculated accordingly. Sampling was conducted in Groningen during the winter (January–February) and summer (June–August) months of 2019. Daily mean temperature ranged from −3°C to 7.8°C, with daily minimum temperatures between −9°C and 2.5°C, and daily maximum temperatures between 0.6°C and 18.3°C in winter. In summer daily mean temperature ranged from 13.8°C to 21.9°C, with daily minimum temperatures between 9.6°C and 15°C, and daily maximum temperatures between 17.3°C and 31.1°C. Over the study period, the range of maximum daily precipitation duration in winter was 11.5–16.6 h, with the range of maximum daily precipitation amounts between 12.7–13.2 (mm). In summer, the range of maximum daily precipitation duration was 4.6–4.9 (h), with the range of maximum daily precipitation amounts between 3.3 and 15.5 (mm). The mean daily sunshine duration was approximately 9 h in winter and 16 h in summer.

### 2.2 DNA Extraction, PCR Amplification, and Amplicon Sequencing

We assessed the diversity of the gut microbiome (16S rRNA amplicon) and diet (metabarcoding) of the feral pigeons using Illumina‐based sequencing. We randomized the gut microbiome and diet metabarcoding samples before DNA extraction.

For the gut microbiome, we extracted genomic DNA from the cloacal swabs using the Powersoil DNA kit (MoBio Laboratories, Carlsbad, CA, USA) according to the manufacturer's instructions apart from two modifications: we added extra sterile glass beads (~0.25 g) to the PowerBead tubes, and cell lysis was achieved by beating all samples three times for 1 min (B. Braun 853022/0 from B. Braun Melsungen AG, Germany, 2820 rpm) instead of a consecutive 3 min to prevent the samples from heating up too much and the DNA was eluted in 100 μL PCR‐grade water. We quantified sample DNA concentrations using nanodrop (Thermo Fisher Scientific). The DNA of 63 feral pigeons and 13 negative control samples was sent to the University of Minnesota Genomic Centre (USA) for sequencing of the V4/V5 region of the 16S rRNA gene in triplicate using forward primer 515 F (5′‐GTGCCAGCMGCCGCGGTAA‐3′) and reverse primer 806 R (5′‐GGACTACHVGGGTWTCTAAT‐3′) (Caporaso et al. 2011) (MiSeq 2 × 300 sequencer, Illumina). PCRs were performed in 10 μL reactions consisting of 5 μL AccuStart 2× PCR master mix (containing Taq DNA polymerase), 1 μL of each primer, 1 μL nuclease‐free water, and 2 μL of DNA. Amplicons were generated using a 25‐cycle PCR with the following settings: 95°C for 5 min; 25 cycles of 98°C for 20 s, 55°C for 15 s, and 72°C for 60 s; followed by 72°C for 5 min and hold at 12°C.

For the diet metabarcoding, we extracted genomic DNA from 0.5 g of the homogenized fecal samples using the PureLink Microbiome DNA Purification Kit (Thermo Fisher Scientific, USA) according to the manufacturer's instructions apart from two modifications: for cell lysis the samples were beated five times for 2 min to prevent the samples from heating up, and the DNA was eluted in 100 μL PCR‐grade water. We used the PureLink kit because in a diet metabarcoding validation study in Pied Flycatchers *Ficedula hypoleuca* , the PureLink kit gave better results than the PowerSoil kit (Verkuil et al. 2022). We included 12 mock diet samples as positive controls (Table S1), and 5 negative control samples (2 for kits, and 3 for PCR plates). We performed the first PCR in triplicate in the lab, using the general metazoan COI primer (mlCOIintF 5′‐GGWACWGGWTGAACWGTWTAYCCYCC‐3′; mlCOIintR: 5′‐GGRGGRTASACSGTTCASCCSGTSCC‐3′) (Leray et al. 2013) and plant ITS2 primer (UniPlantF: 5′‐TGTGAATTGCARRATYCMG‐3′; UniPlantR: 5′‐CCCGHYTGAYYTGRGGTCDC‐3′) (Moorhouse‐Gann et al. 2018), using this thermal cycling protocol: 3 min at 94°C, 35 cycles with 60 s at 94°C, 30 s at 48°C for metazoan COI primer or 30 s at 56°C for plant ITS2 primer, 60 s at 72°C, followed by 10 min at 72°C. PCRs were performed in 10 μL reactions consisting of 5 μL AccuStart 2× PCR master mix (containing Taq DNA polymerase), 1 μL of each primer, 1 μL nuclease‐free water, and 2 μL of DNA. We pooled the triplicates after the PCR and checked using 4 μL of PCR product with gel electrophoresis. The library was prepared using the MiSeq V3 kit (Illumina) and sequenced with 300‐bp paired‐end reads on a MiSeq sequencer at the Department of Human Genetics, University Medical Centre, Leiden, The Netherlands, aiming for 50,000 raw reads per sample.

### 2.3 Mock Diets

We used mock diet samples as positive controls to determine if the read count reflects original biomass after sequencing. We composed mock diet samples by including two of the following four potential diet components: two human‐processed food items commonly eaten by feral pigeons, bread (wheat) and franch fries (potatoes), and two natural plant species, dandelion *Taraxacum* and peas *Pisum*. The two food items (four combination variants) were included in three different proportions of dry mass (9:1, 5:5, 1:9), making a total of 12 mock diet samples (Table S1). Because the food items were expected to differ in dry mass, we first determined water content by drying a portion of the materials to constant mass (SuppInfo: Drying procedure). We set the total mass of each mock sample to 0.2 g dry mass and calculated the required wet mass for each diet item based on its water content (Table S1). All items were grinded by pestle and mortar, mixed proportionally, and stored in a 2 mL centrifuge tube in a 4°C refrigerator until DNA extraction.

All four mock diet food items were present in the sequence results, thus also the two human‐processed food items. We compared the actual proportions with the expected proportions for each mock sample (Figure S1). This showed that in only one mock diet sample (M6) the actual proportions of peas and potatoes met the expectations. Potatoes and wheat exhibit a clear decline in relative abundance in all other samples, especially when the contents of these crops are low (M1–M5, M10 and M12). We assume that this is due to DNA damage from the deep processing of human foods (fries and bread). This suggests that using relative read abundances will lead to an underestimation of the contribution of potatoes and wheat to the diet, and thus an overestimation of other diet items. Peas and dandelions yielded more sequences, but for these food items the actual proportions did not match expectations (M7–M9), possibly due to difficulties in mixing those two materials well. In M10–M12, peas appear to have been present as contaminants. Given these results, we choose to analyze diet data using frequency of occurrence of reads instead of using relative read abundance (Deagle et al. 2019).

### 2.4 Sequence Preprocessing and Bioinformatics Approaches

For the gut microbiome, we processed the raw sequence data using the standard QIIME2 protocol (v2021.4) (Bolyen et al. 2019). Using the DADA2 (v2021.4) pipeline, we trimmed the primers, truncated the forward and reverse reads with 19 and 20 bp, respectively at the 5′ end of the input sequences, and truncated the reverse reads 260 bp at the 3′ end of the input sequences due to low quality. The taxonomy table was built using the Silva v138 reference database (Quast et al. 2013). Next, we removed sequences that showed less than 50% identity to the SILVA v138 reference database, using a BLAST‐based quality‐control step. We then filtered out sequences assigned to mitochondria, chloroplasts, and Archaea based on taxonomic classification prior to downstream analyses. Amplicon Sequence Variant (ASV) table and phylogenetic tree were processed in R (v4.4.0) using the phyloseq (v1.48.0) package. We first identified and removed contaminations from the data using the package decontam (v1.24.0) with the prevalence method. With this method, 31 out of 1116 ASVs were flagged as contaminants, most of which showed high read counts in negative controls and low counts in true samples.

The richness rarefaction curves leveled off at 3000 read counts, hence we rarefied the data to the number of read counts of the sample just above 3000 read counts. Hereafter, the data included 981 taxa divided over 59 feral pigeon samples, with 194,405 read counts. The median proportion of reads unassigned at the phylum level per sample was 1.5%.

For diet metabarcoding, we used cutadapt (v4.4) (Martin 2011) and unoise3 in Usearch (v11) (Edgar 2010) to cut primers, dereplicate reads, and transform the raw Illumina sequence data into a list of ASVs. Using the BLASTn algorithm, ASV sequences were matched to the nucleotide database in GenBank (National Centre for Biotechnology Information (NCBI); using a local copy created on April 3rd, 2024; Benson et al. 2012) and the Barcode of Life Database (BOLD, using a local copy created on March 11th, 2024; Ratnasingham and Hebert 2007). The NCBI database contains reference sequences of taxa from all domains of life (Benson et al. 2012), and BOLD contains mostly cytochrome c oxidase I (COI) sequences of Metazoan (Ratnasingham and Hebert 2007).

Taxonomic assignment of BLAST results was conducted with the Lowest Common Ancestor (LCA) method implemented in the galaxy‐tool‐lca (https://github.com/naturalis/galaxy‐tool‐lca) workflow (Huson et al. 2007; Beentjes et al. 2019), considering the top 8% hits based on bitscore, with a minimum identity of 80% and coverage of 80%, and using a cut‐off of 98% sequence identity and 100% coverage for species‐level assignments. In case of more than one species‐level assignment, an ASV was assigned to the LCA of all species‐level assignments. Conflicting assignments were resolved at the highest shared taxonomic level, with kingdom‐level discrepancies prioritized according to NCBI taxonomy. Top hits were accepted when consistent with the other database's LCA, and when both databases provided only top hits, consensus was based on their lowest common ancestor. ASVs without reliable matches in either database were classified as unidentified. We included 3 PCR negative controls, 2 kit negative controls, and 8 feces negative controls. For each ASV, we recorded the highest read count observed across all negative controls and excluded this ASV from any sample where its read count did not exceed this threshold (Verkuil et al. 2022). The richness rarefaction curves leveled off at 1000 read counts; hence, we considered this sequencing depth sufficient to capture the majority of taxonomic diversity in the metabarcoding dataset.

### 2.5 Statistical Analysis

#### 2.5.1 Host Traits

We used linear mixed models (LMM; nlme package (v 3.1.167)) to identify correlations between the host traits (body mass, wing length, and head‐bill length) and season (summer vs. winter). We used season, sex and their interaction in the initial model and we included bird ID as random effect. Visual inspection of residual plots revealed no apparent deviations from homoscedasticity or normality. *p* values were obtained by ANOVA tests of the full model with the effect in question against the model without the effect in question.

#### 2.5.2 Alpha‐Diversity of the Gut Microbiome

Alpha diversity metrics and beta diversity metrics were calculated by using the packages phyloseq (v1.48.0) and btools (v0.0.1). For alpha‐diversity indices (Observed richness, Shannon index, and Faith's phylogenetic diversity), we tested the effects of season, location, and their interaction. Faith's PD was included as a phylogeny‐based complement to ASV richness (Faith 1992). Because the distributions of these indices deviated from normality, and bird ID did not explain variation in any of the indices, we employed generalized linear models (GLM). We used a stepwise backward exclusion of nonsignificant fixed factors and checked the normality of the model and homoscedasticity of the residuals at each step of the analysis.

#### 2.5.3 Gut Microbiome Community Composition Differences

The composition of the gut microbiome community (beta‐diversity) was evaluated by examining the taxonomic similarities across seasons and locations. This was done using the Jaccard similarity index, Bray–Curtis dissimilarities, and both unweighted and weighted UniFrac distances; they allow us to assess whether patterns are driven by taxon turnover, changes in dominant taxa, or phylogenetic shifts (Lozupone and Knight 2005). To assess whether community clustering and group dispersion varied by season or location, we conducted a principal coordinate ordination analysis (PCoA) on beta‐diversity indices. This involved modeling the similarities and distances derived from an ASV level table using Permutational Multivariate Analysis of Variance using Distance Matrices (PERMANOVA), supported by 999 permutations (McArdle and Anderson 2001). PERMANOVA analyses were conducted using the adonis2() function in the vegan package (v2.6.10). Subsequently, the impact of season and location, as well as their interaction, was analyzed. Prior to each analysis, we tested for the influence of individual (bird ID) as repeated measures were available. When bird ID was significant, it was included as a blocking factor in the permutation test. Additionally, we examined the homogeneity of dispersion (Anderson 2006) for each predictor. Predictors associated with significant dispersion effects were excluded from the final models.

We fitted generalized linear models (GLM) to examine variation in the relative abundances of the most abundant phyla (top four) and genera (top four). As fixed variables, we included season, location, and their interaction term. Before running the GLMs, taxa proportions were logit transformed as log [(*p* + *e*)/(1 − *p* + *e*)], where p is the proportion of taxa in a given sample and *e* is the lowest proportion (among samples) for that taxon excluding zero (Warton and Hui 2011).

#### 2.5.4 Diet Analyses

We used the frequency of occurrence (FOO) of each family to provide insight into the prevalence of each dietary component in the diet of the feral pigeons. The FOO was calculated per family diet ASV as FOO = (*n*/*t*) × 100, where *n* is the number of samples in which the family was detected and *t* is the total number of samples.

We applied Non‐metric Multidimensional Scaling (NMDS) to demonstrate variances in diet compositions. NMDS condenses complex multi‐dimensional data into NMDS dimensions based on rank orders, simplifying both the visualization and the interpretation of the data (Faith et al. 1987; Minchin 1987). We performed NMDS on presence/absence data using the Jaccard distance and evaluated ordination fit using the stress value (< 0.1 excellent, < 0.2 good). PERMANOVA and tests of multivariate homogeneity of group dispersions were also conducted.

#### 2.5.5 Impact of Diet, Environment and Host Traits on the Gut Microbiome

We used Procrustes analysis to compare diet (Jaccard) and gut microbiome (Jaccard) PCoA ordinations, with significance assessed by 9999 permutations. We assessed associations between microbiome community composition and explanatory variables using distance‐based redundancy analysis (dbRDA) implemented with the capscale(). The full dbRDA model included temperature (Temp_PC1), precipitation (Prec_PC1), location (Location), diet (Diet_PCoA1 and Diet_Observed), and host traits (BCI and Sex). Daylength was not included because it was highly collinear with temperature across the two seasons. Significance of marginal effects of predictors was evaluated using permutation tests (999 permutations). To test whether temperature microbiome associations were robust to spatial structure, we fitted a partial dbRDA with location included as a conditioning variable. All figures were plotted by using ggplot2 package (v4.0.2). For completeness, we report the PLS‐PM methods and results in Appendix Information: PLS‐PM (Tables S2 and S7).

## 3 Results

### 3.1 Seasonal Variation in Host Traits

Feral pigeons were heavier in winter (336 ± 5.6 g) than in summer (312 ± 5.2 g; *F*_*1,6*_ = 15.3, *p* = 0.01), and males (339 ± 5.4 g) were heavier than females (309 ± 7.1 g, *F*_*1,48*_ = 11.7, *p* = 0.001). Wing length and head bill length did not vary between summer and winter, but males had larger wings (238 ± 0.9 mm, *F*_*1,50*_ = 24.23, *p* < 0.0001) and head bill lengths (54.2 ± 0.2 mm, *F*_*1,50*_ = 4.55, *p* = 0.04) than females (wing: 230 ± 1.3 mm, head bill: 53.4 ± 0.3 mm, *p* = 0.04).

### 3.2 Gut Microbiome Community Diversity and Composition

**FIGURE 1:**
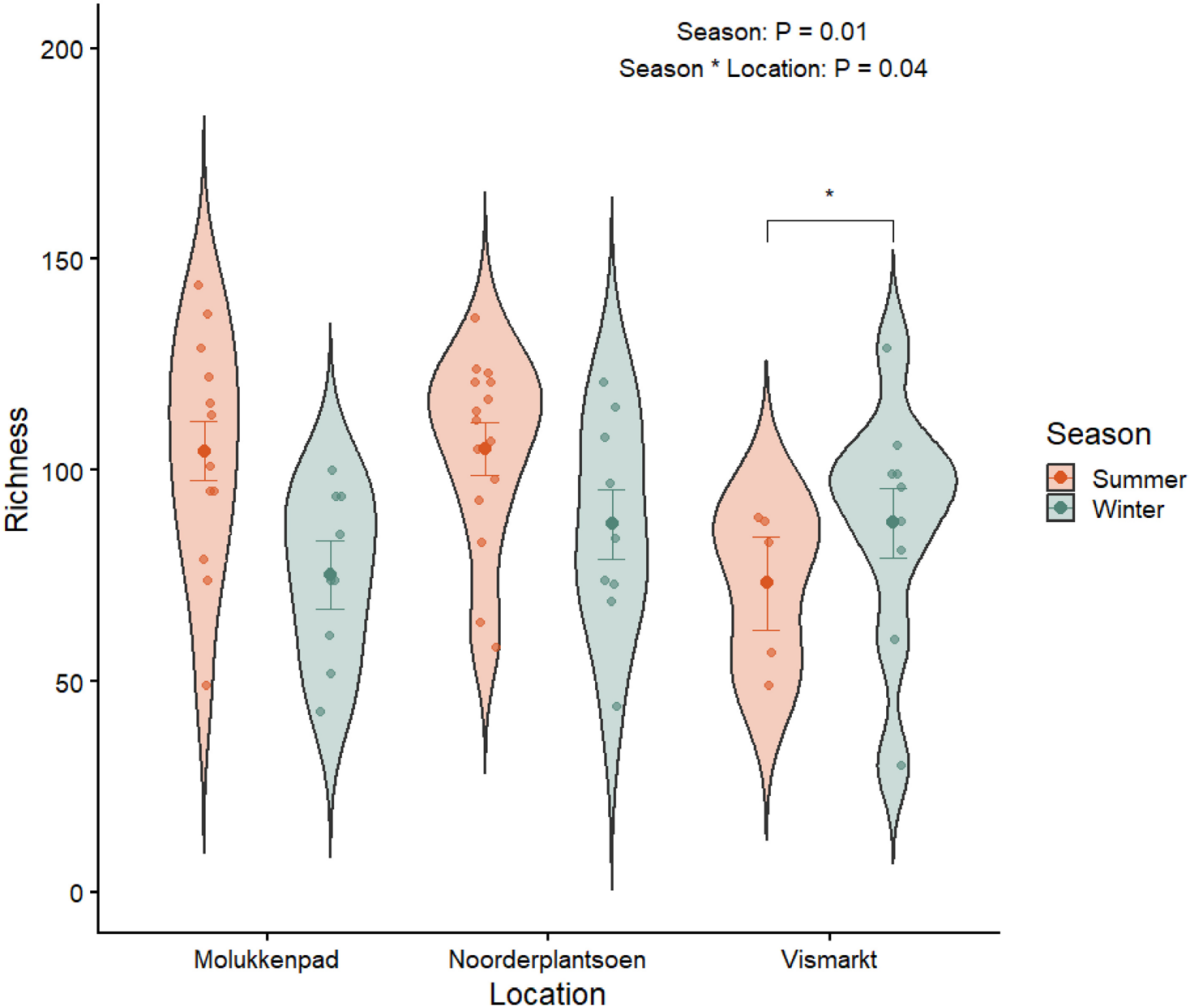
The richness of the gut microbiome across seasons and locations. Violin plots show the distribution of observed richness with individual samples (jittered points). Colored points with error bars represent GLM‐predicted means (± SE). A significant interaction between season and location was detected (*p* = 0.04). Urban site (Vismarkt) is shown in red and suburban sites (Noorderplantsoen and Molukkenpad) in green. (summer: *N* = 33, winter: *N* = 30; Molukkenpad: *N* = 23, Noorderplantsoen: *N* = 26, Vismarkt: *N* = 14).

For alpha diversity, we found that richness was significantly lower in winter compared to summer (Figure 1; Table S3). The effect of season on richness also varied by location (Figure 1), with a significant interaction at the Vismarkt site, where richness was higher in winter than in summer (post hoc test: *p* = 0.02). No significant effects of season or location were detected for Shannon diversity or Faith's PD (Table S3).

**FIGURE 2:**
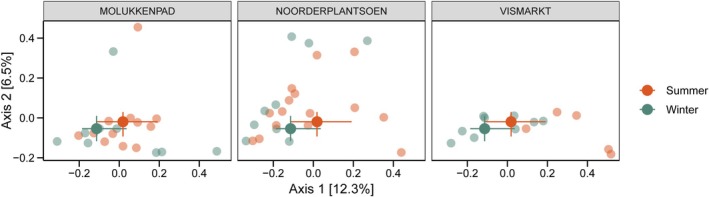
Seasonal variation in Jaccard distances visualized by PCoA plots per location. Large symbols represent group medians, with error bars indicating the interquartile range (25%–75%). Smaller points represent individual samples. Axis 1 and Axis 2 explain 12.3% and 6.5% of the variation, respectively. Vismarkt is an urban site and Noorderplantsoen and Molukkenpad are suburban sites (Summer: *N* = 33, winter: *N* = 30; Molukkenpad: *N* = 23, Noorderplantsoen: *N* = 26, Vismarkt: *N* = 14).

Season and location contributed to the variation in microbial community composition, but this varied between different indexes of beta‐diversity. For Jaccard distance, significant effects of both season and location were detected, with no evidence of an interaction between the two factors (Figure 2; Table S4), and PCoA1–2 together explained 18.8% of the variation. Bray–Curtis distances similarly indicated a significant seasonal effect (Table S4). However, because the test of homogeneity of multivariate dispersion showed significant differences in dispersion among locations (*p* = 0.047), and PERMANOVA can be sensitive to heterogeneous dispersion, location was not retained in the final model. Unweighted UniFrac distances revealed significant variation among locations, but there was no seasonal effect (Table S4). Weighted UniFrac distances showed no significant variation with either season or location (Table S4).

#### 3.2.1 Seasonal Variation in the Relative Abundance of Dominant Phyla and Genera in the Gut Microbiome

The taxa were divided over 13 phyla. We selected the four phyla with the highest relative abundances to test for variations with season, location, and their interaction. The top four phyla were Firmicutes (43.1% ± 17.7%), Actinobacteria (26.0% ± 0.1%), Proteobacteria (13.1% ± 0.1%), and Tenericutes (5.5% ± 0.1%). The GLM analysis revealed significant effects for some factors on the logit‐transformed proportions of dominant phyla (Table S5). In summer, Actinobacteria showed a significantly lower proportion compared to winter, while location did not show significant effects (Table S5). In Tenericutes, the proportion was significantly higher at Noorderplantsoen than at the other locations, while the effect of season was not significant (Table S5). For Firmicutes and Proteobacteria, neither the main effects of season, location, nor their interactions were statistically significant (Table S5).

The four most abundant genera were *Lactobacillus* (16.6% ± 13.9%), which included the most abundant ASV, *Enterococcus* (10.9% ± 15.9%), *Corynebacterium* (7.5% ± 4.5%), and *Shigella* (5.8% ± 11.1%). Using the same initial model as for the phyla, we found that the logit proportion of *Lactobacillus* was lower in winter compared to summer, while the effects of location were not significant (Table S6). The logit proportion of *Enterococcus* was also lower in winter than in summer (Table S6). In addition, the interaction between season and location was significant, indicating a lower proportion of *Enterococcus* at Vismarkt during winter (Table S6). The logit proportion of *Corynebacterium* varied with location (Table S6); post hoc pairwise comparisons found a difference between Noorderplantsoen and Vismarkt (estimate: 0.4 ± 0.1SE, *p* = 0.005), but not between Molukkenpad and Noorderplantsoen (−0.2 ± 0.1SE, *p* = 0.24) or between Molukkenpad and Vismarkt (0.2 ± 0.1SE, *p* = 0.18). None of the season or location effects reached statistical significance in *Shigella* (Table S6).

### 3.3 Metazoan Components of the Diet

Filtering the initial 465 metazoan (multicellular animals) ASVs (removing unidentified ASVs, singletons, and zero‐read features) yielded 288 ASVs belonging to nine phyla: Arthropoda, Annelida, Mollusca, Cnidaria, Polychaeta, Chordata, Nematoda, Platyhelminthes, and Rotifera. The sequences from Nematoda, Platyhelminthes, and Rotifera were common bird parasites (McDougald 2020) and therefore removed from the data, as well as sequences from Chordata, leaving 110 ASVs in five phyla. The feral pigeon diet included three families and one order of Mollusca (*Agriolimacidae*—small and medium‐sized land slugs, *Camaenidae*, *Hygromiidae*—land snails, Cardiida—clams) in 17 samples. One genus of Cnidaria (*Helgicirrha*–hydrozoans), one species of Annelida ( *Lumbricus rubellus* ), and Polychaeta were found in only 7 samples. Next, we excluded the Cnidaria, Polychaeta, and Hexanauplia ASVs because they are marine organisms and thus unlikely eaten by feral pigeons. The vast majority of the final 102 identified metazoan ASVs of the diet belonged to the Arthropoda, including Insecta (insects), Arachnida (spiders and mites), and Collembola (springtails). Metazoan reads accounted for 2.6% of the total sequences generated with the COI primer set. Because the COI primers also amplified substantial non‐target taxa, resulting in a low fraction of reads assigned to metazoan, and the same pattern was also observed in other studies (Gutiérrez‐Galán et al. 2017; Schumm et al. 2023). Given the low proportion of target metazoan reads and the resulting limited power for quantitative comparisons, we excluded the metazoan component from downstream diet analyses and focused on the plant component.

### 3.4 Plant Components of the Diet

From 1194 detected plant ASVs, removal of unidentified, zero‐read, and singleton ASVs reduced the dataset to 504 ASVs, representing 97.4% of total reads. We found one Pinophyta species, *Taxus baccata* , commonly known as the European yew. All other ASVs were from the Streptophyta, including the classes Klebsormidiophyceae (*Klebsormidium*, green algae) and Magnoliopsida (flowering plants). Magnoliopsida included 26 orders, 41 families, and 96 genera. A total of 146 ASVs were detected in summer and 110 ASVs were detected in winter. The diet of the feral pigeons at Noorderplantsoen included 119 plant ASVs, at Molukkenpad 104 ASVs, and at Vismarkt 79 ASVs.

**FIGURE 3:**
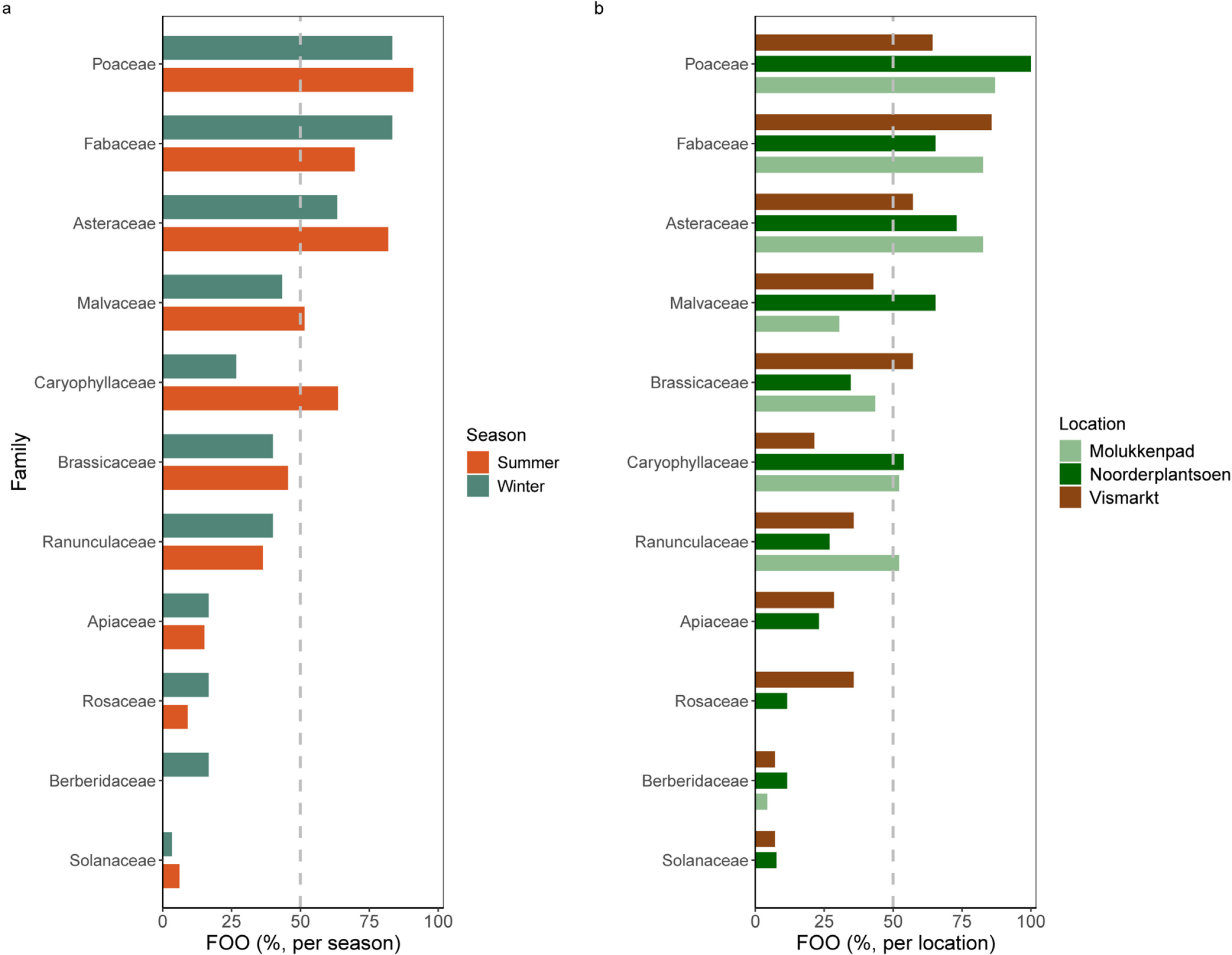
Bar plots showing the frequency of occurrence (FOO) of the most frequently detected plant families in the diet of feral pigeons across (a) seasons and (b) locations. Dashed lines indicate 50% occurrence within each group (summer: *N* = 33, winter: *N* = 30; Molukkenpad: *N* = 23, Noorderplantsoen: *N* = 26, Vismarkt: *N* = 14). Urban site (Vismarkt) is shown in brown and suburban sites (Noorderplantsoen and Molukkenpad) in green.

We did not find an effect of season (estimate: –7.2 ± 3.9SE, *p* = 0.12; Figure S2) nor location (Noorderplantsoen: 5.2 ± 4.5SE, *p* = 0.25; Vismarkt: –3.9 ± 5.3SE, *p* = 0.47; Figure S2) on the diet prevalence of plant ASVs (FOOs). We did find indications that the feral pigeon's diet included anthropogenic food items, namely potato ( *Solanum tuberosum* ), but the vast majority of the ASVs belonged to families of natural plants: *Poaceae*, *Brassicaceae*, *Caryophyllaceae*, *Asteraceae*, *Fabaceae*, *Malvaceae*, *Rosaceae*, *Ranunculaceae*, *Berberidaceae*, *Solanaceae* and *Apiaceae*. Among these, the families *Poaceae*, *Fabaceae* and *Asteraceae* had the highest prevalence (FOO > 50% of the samples) in both seasons and at all locations (Figure 3). Within these three families, we did find variation in ASV prevalence with season and location. *Triticum* spp. (grass) and *Poa* spp. (meadow‐grass) had a higher prevalence in summer than in winter. *Triticum* spp. had a higher prevalence at Noorderplantsoen, while *Poa* spp. and *Pisum* spp. (pea) had a higher prevalence at Molukkenpad (Figure S3).

#### 3.4.1 Plant Diet Community Composition Analysis Using NMDS

Using NMDS, we did not find a significant effect of season on plant ASV presence/absence at the taxon level, but there was an effect of location (Figures S4 and S5). At both family and genus level, we found presence/absence differences between locations (family: *k* = 3, stress = 0.1, *p* = 0.009, Figure S4; and genus *k* = 3, stress = 0.1, *p* = 0.001, Figure S5), namely location Molukkenpad differed from location Noorderplantsoen (permutation test: *F*_*1,61*_ = 2.06, *p* = 0.01, and *F*_*2,60*_ = 2.93, *p* = 0.003, for family and genus level, respectively).

#### 3.4.2 Impact of Plant Diet, Environment and Host Traits on the Gut Microbiome

**FIGURE 4:**
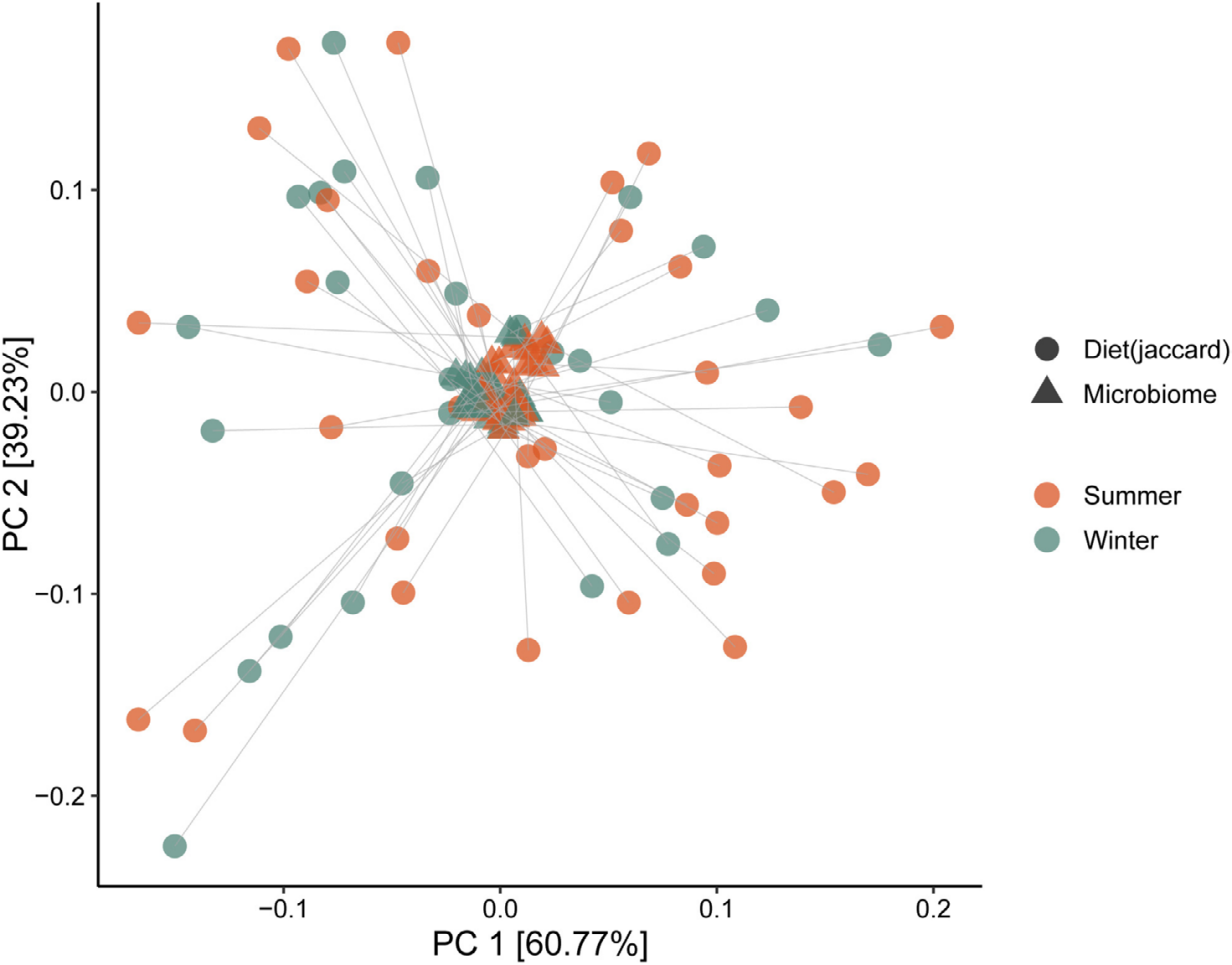
Procrustes analysis of diet composition and gut microbiome composition based on PCoA. Solid lines connect the same individual. Different shapes indicate different types of samples. Different colors indicate different seasons. Percentages on the axes show the variation explained. Effective sample size (*n* = 59).

Procrustes analysis showed no significant relationship between plant diet and gut microbiome composition, nor was there any clear seasonal separation at genus level (*p* = 0.63; Figure 4).

Using Jaccard distances (presence/absence), distance‐based redundancy analysis (dbRDA) identified temperature (Temp_PC1), location, and sex as significant predictors of gut microbiome composition (Table S8). In contrast, precipitation (Prec_PC1), diet (Diet_PC1 and Diet_Observed), and body condition (BCI) were not significant (Table S8). When controlling sampling location, the association with temperature remained significant (Table S8), whereas diet remained non‐significant.

**FIGURE 5:**
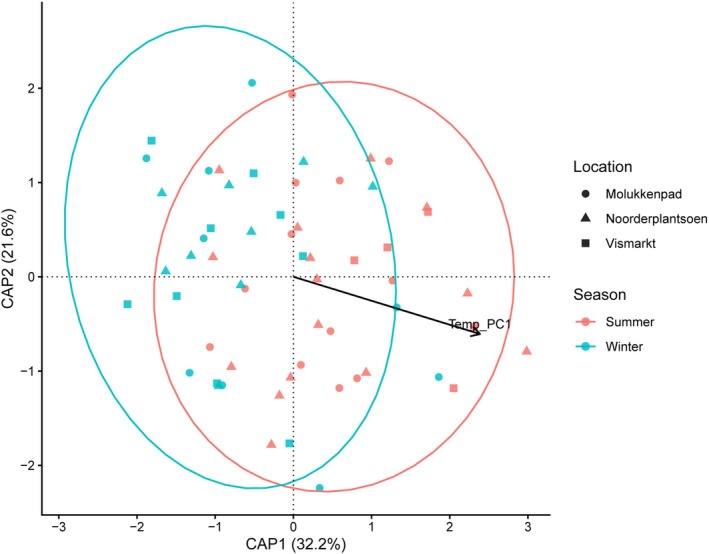
Distance‐based redundancy analysis (dbRDA) of cloacal microbiome composition based on Jaccard distances. Points are colored by season and shaped by location; ellipses indicate 95% confidence intervals around seasonal centroids. The arrow shows the fitted direction of increasing temperature (Temp_PC1) in the constrained ordination space. Percentages on CAP axes represent the proportion of constrained variation explained by each axis. Vismarkt is an urban site and Noorderplantsoen and Molukkenpad are suburban sites (Summer: *N* = 33, winter: *N* = 30; Molukkenpad: *N* = 23, Noorderplantsoen: *N* = 26, Vismarkt: *N* = 14).

In the constrained ordination, samples showed substantial overlap between seasons, consistent with high interindividual variability, but the fitted temperature vector aligned with the main constrained axis (Figure 5). Variation partitioning supported the same patterns. The unique fractions attributable to the environmental predictor and location were significant, whereas the diet was not (Table S9).

## 4 Discussion

This study provides new insights into seasonal variation in the gut microbiome of feral pigeons, highlighting the complex interplay between diet, environmental factors, and host body condition. Our results show that the gut microbiome composition of feral pigeons differs across seasons, reflecting shifts that coincide with seasonal environmental variation. Importantly, our findings did not reveal clear seasonal shifts in diet, suggesting that the measured diet variation is unlikely to explain the seasonal microbiome variation and that environmental variables were more strongly associated with microbiome composition.

In many wild bird systems, seasonal microbiome variation is often reported to coincide with seasonal dietary shifts, which provides a useful contrast for interpreting our results. For example, changes in the diet of thick‐billed murres ( *Uria lomvia* ) across seasons and between sexes, which were linked to changes in their gut microbial communities (Góngora et al. 2021). Similarly, the gut microbiota of Sichuan partridges ( *Arborophila rufipectus* ) exhibited clear seasonal fluctuations that corresponded to shifts in their diet (Tang et al. 2023). Review studies further reinforce this pattern, suggesting that among various ecological and intrinsic factors—such as phylogeny, habitat, nesting environment, and season—diet is consistently identified as the most influential determinant of gut microbiota composition (Matheen et al. 2022; Uehling and Houtz 2025). However, this strong emphasis on diet raises an important question: are seasonal changes in gut microbiota necessarily dependent on dietary shifts? The results from dbRDA and variation partitioning analyses indicated significant independent contributions of temperature and location, whereas diet did not explain microbiome seasonal variation. Several studies have similarly reported that gut microbial communities can shift rapidly with temperature and environmental heterogeneity (Chevalier et al. 2015; Videvall et al. 2025). This suggests that seasonal changes in the gut microbiota of urban birds are not fully accounted for by diet alone and may instead reflect seasonal environmental conditions.

Two non‐mutually exclusive hypotheses could explain these associations. One possible explanation is the hypothesis that the environment serves as a direct microbial source, with microorganisms from soil, air, and water potentially entering the gut during feeding or drinking (Bodawatta et al. 2022; Grond et al. 2018; van Veelen et al. 2018). Another hypothesis is that environmental variation may act indirectly by altering host physiology, for instance through seasonal changes in immune activity, hormone levels, and energy expenditure which are known to correlate with gut microbiome composition (van Veelen et al. 2020; Zhu et al. 2022; Chevalier et al. 2015). Importantly, seasonal environmental effects need not operate uniformly across individuals or sites, and may be modulated by fine‐scale habitat heterogeneity and host‐specific factors. Recent work in wild birds has shown that pronounced seasonal patterns often persist alongside strong differentiation within populations and within seasons, linked to microhabitat heterogeneity and individual‐level effects (Videvall et al. 2025). Likewise, studies on estrildid finches demonstrate that individual‐specific microbial signatures can remain stable despite shared environments (Maraci et al. 2021), and broader comparative analyses suggest that seasonal and environmental drivers may interact with host identity rather than acting in isolation (Liukkonen et al. 2024).

Unlike many wild birds, feral pigeons not only consume natural foods such as plants, but may also rely heavily on human food sources—including processed grains, bread, and general food waste—which are widely available in urban environments, and may affect the gut microbiome (Murton and Westwood 1966; Lim et al. 2023). The Vismarkt location, situated in the city center, provides pigeons with particularly easy access to human‐derived foods. Consistent with this, we detected plant taxa from Solanaceae (e.g., potato) in the diet from both Vismarkt and Noorderplantsoen. These urban food resources are typically higher in carbohydrates and fats and remain stable across seasons (Birnie‐Gauvin et al. 2017), which may help explain the limited seasonal variation in diet we observed. However, the stability of urban food supply does not necessarily prevent seasonal microbiome shifts. In Vismarkt, for instance, we found higher gut microbiome richness in winter than in summer.

The seasonal patterns in the gut microbiome of free‐living feral pigeons can be further interpreted by comparing our findings with those from homing pigeons studied under controlled dietary conditions. Dietz et al. (2022) demonstrated that homing pigeons show clear summer–winter differences in gut microbiome composition even when diet remains constant, with higher abundances of Firmicutes in winter and higher Bacteroidetes in summer. In contrast to this pattern, urban feral pigeons in our study showed decreases in two Firmicutes genera, *Lactobacillus* and *Enterococcus*, during winter, despite similarly stable diets. These differences suggest that while both studies demonstrate that seasonal gut microbiome changes can occur independently of diet, the direction and identity of the shifting taxa differ depending on environmental context.

While our findings highlight the potential for environmental variation associate with microbiome seasonality in urban birds, important limitations remain. Without physiological or immune data and information about environmental microbial communities, we cannot determine whether the observed microbiome shifts are mediated by host responses or direct microbiome colonization from the environment. Environmental variables were derived from a single weather station and sample sizes were uneven across sites, which may mask fine‐scale urban heterogeneity and contribute to location‐specific effects. A further limitation concerns about the microbiome was quantified from cloacal swabs, which likely reflect a gut‐associated community but may also integrate signals from the distal gut, the urogenital tract, whereas diet was inferred from fecal samples, representing a different biological compartment and temporal window. In addition, feral pigeons may feed on different parts of plants (e.g., seeds, leaves, flowers) across seasons which differ in their nutritional composition. Seeds are typically rich in carbohydrates and fats, while leaves and flowers contain more fiber, proteins, and secondary metabolites (Lykke and Padonou 2019; Borecka and Karas 2025). Such dietary variation may influence gut microbiome composition in ways not captured by our metabarcoding approach. Pigeons may also be exposed to human‐associated microbes via anthropogenic food waste, although we did not measure this pathway and its importance remains unknown.

These limitations highlight several opportunities to strengthen inference in future work. In particular, considering populations with more clearly differentiated diets or contrasting environments, extending monitoring across multiple years, and incorporating high‐resolution dietary (microscopic examination and trying different primer pairs), environmental microbiome, and host physiological data will help place seasonal microbiome dynamics in a broader ecological context. Against this backdrop, our results offer a clear example of how environmental seasonality can be associated with gut microbiome dynamics in urban wildlife.

## 5 Conclusion

Our study reveals that the gut microbiome of feral pigeons exhibits distinct seasonal shifts in urban environments, despite the absence of pronounced dietary seasonality. This indicates that environmental conditions—rather than diet alone—can be associated with seasonal changes in gut microbiome communities. By demonstrating that urban birds experience microbiome seasonality even under a relatively stable diet, our findings expand current understanding of the factors shaping avian gut microbiomes and underscore the importance of environmental seasonal variation as a key ecological force.

## Author Contributions

**Kangqing Zhang:** conceptualization (equal), data curation (equal), formal analysis (lead), funding acquisition (supporting), investigation (equal), methodology (lead), software (lead), validation (lead), visualization (lead), writing – original draft (lead), writing – review and editing (lead). **Henri Zomer:** conceptualization (supporting), data curation (equal), investigation (equal), methodology (supporting). **Gerrit Potkamp:** data curation (supporting), formal analysis (supporting), investigation (supporting), methodology (supporting), writing – review and editing (supporting). **Joana Falcao Salles:** formal analysis (supporting), software (supporting), supervision (equal), writing – review and editing (equal). **B. Irene Tieleman:** conceptualization (equal), funding acquisition (supporting), project administration (equal), resources (equal), supervision (equal), writing – review and editing (equal). **Maurine W. Dietz:** conceptualization (lead), data curation (supporting), formal analysis (supporting), funding acquisition (lead), investigation (supporting), methodology (supporting), project administration (lead), resources (lead), software (supporting), supervision (lead), writing – review and editing (equal).

## Funding

This work was supported by the China Scholarship Council (CSC).

## Disclosure

Statement on Inclusion: Our study brings together authors from different backgrounds, including animal ecologists, microbial ecologists, and bioinformatic scientists, but also different origins, namely Dutch, Chinese, and Brazilian. All authors were engaged early on with the research and study design to ensure that the diverse sets of perspectives they represent were considered from the onset. This study is part of a PhD project on seasonal variation in the gut microbiome in temperate and tropical birds, in which we will strengthen our collaborations with African countries such as Kenya.

## Conflicts of Interest

The authors declare no conflicts of interest.

## Supporting information


**Table S1:** The water content and weight of each material in diet mock samples.
**Table S2:** Outer model specifications for the Partial Least Squares Path Modeling (PLS‐PM) analysis, including latent variables, their corresponding manifest variables, data processing procedures, and the proportion of variance explained.
**Table S3:** Results of factorial ANOVA assessing the main and interactive effects of Season and Location on multiple alpha diversity metrics, including richness, Shannon diversity, and Faith's phylogenetic diversity.
**Table S4:** Permutational multivariate analysis of variance (PERMANOVA) results showing the effects of Season, Location, and their interaction on multiple beta diversity metrics (Jaccard, Bray–Curtis, Unweighted UniFrac, and Weighted UniFrac).
**Table S5:** Generalized linear model (GLM) results testing the effects of season, location, and their interaction on the logit‐transformed relative abundances of the four dominant bacterial phyla.
**Table S6:** Generalized linear model (GLM) results testing the effects of season, location, and their interaction on the logit‐transformed relative abundances of the four dominant bacterial genera.
**Table S7:** Sensitivity of PLS‐PM path coefficients to alternative measurement specifications (bootstrap percentile 95% CI, 5000 resamples).
**Table S8:** Distance‐based redundancy analysis (dbRDA) of gut microbiome composition identifies independent effects of temperature, location, and sex.
**Table S9:** Unique contributions of environmental, diet, host, and location variables were tested using partial redundancy analysis (partial RDA) with permutation tests.
**Figure S1:** Comparison of the expected proportions (EX) of four taxa (Fabaceae, peas; Solanaceae, potato; Taraxacum, dandelion; Triticum, wheat) versus the actual proportions (RE) in mock samples. EX shows the proportions of the dry mass of the two taxa for each mock sample (see Table S1). RE shows the proportions of the actual relative abundances of the taxa obtained in each mock sample after sequencing. Different colors indicate different taxa.
**Figure S2:** Violin plot showing the overall differences in richness of the diet of feral pigeons between (a) seasons and (b) locations. Dots represent individual samples, while the box show the median, interquartile range, and variability of the data (Summer: *n* = 33, winter: *n* = 30; Molukkenpad: *n* = 23, Noorderplantsoen: *n* = 26, Vismarkt: *n* = 14).
**Figure S3:** Frequency of occurrence (FOO) of various plant genera consumed by feral pigeons in different (a) seasons and (b) locations. The dashed lines indicate that the genus was found in more than half of the total samples per season (summer: 33, winter: 30) or location (Molukkenpad: 23, Noorderplantsoen: 26, Vismarkt: 14).
**Figure S4:** NMDS plots showing patterns of diet composition of feral pigeons across season (a–c) and locations (d–f) at family level. The ellipses show the 95% confidence regions, indicating the clustering of samples of similar groups (Summer: *n* = 33, winter: *n* = 30; Molukkenpad: *n* = 23, Noorderplantsoen: *n* = 26, Vismarkt: *n* = 14).
**Figure S5:** NMDS plots showing patterns of diet composition of feral pigeons across between season (a–c) and locations (d–f) at genus level. The ellipses show the 95% confidence regions, indicating the clustering of similar samples (Summer: *n* = 33, winter: *n* = 30; Molukkenpad: *n* = 23, Noorderplantsoen: *n* = 26, Vismarkt: *n* = 14).

## Data Availability

The datasets supporting this article are available in DataverseNL (https://doi.org/10.34894/9UNDGT). These include the raw sequences, metadata, and R scripts used in this study.

## References

[ece373682-bib-0001] Anderson, M. J. 2006. “Distance‐Based Tests for Homogeneity of Multivariate Dispersions.” Biometrics 62, no. 1: 245–253. 10.1111/j.1541-0420.2005.00440.x. 16542252

[ece373682-bib-0002] Bailey, M. T. , J. C. Walton , S. E. Dowd , Z. M. Weil , R. J. Nelson , and J. P. Williams . 2010. “Photoperiod Modulates Gut Bacteria Composition in Male Siberian Hamsters ( *Phodopus sungorus* ).” Brain, Behavior, and Immunity 24, no. 4: 577–584. 10.1016/j.bbi.2009.12.010. 20045457

[ece373682-bib-0003] Beentjes, K. K. , A. G. C. L. Speksnijder , M. Schilthuizen , M. Hoogeveen , R. Pastoor , and B. B. van der Hoorn . 2019. “Increased Performance of DNA Metabarcoding of Macroinvertebrates by Taxonomic Sorting.” PLoS One 14, no. 12: e0226527. 10.1371/journal.pone.0226527. 31841568 PMC6913968

[ece373682-bib-0004] Benson, D. A. , M. Cavanaugh , K. Clark , et al. 2012. “GenBank.” Nucleic Acids Research 40: D48–D53. 22144687 10.1093/nar/gkr1202 PMC3245039

[ece373682-bib-0005] Birnie‐Gauvin, K. , K. S. Peiman , D. Raubenheimer , and S. J. Cooke . 2017. “Nutritional Physiology and Ecology of Wildlife in a Changing World.” Conservation Physiology 5, no. 1: cox030. 10.1093/conphys/cox030. 28740638 PMC5516125

[ece373682-bib-0006] Bodawatta, K. H. , S. M. Hird , K. Grond , M. Poulsen , and K. A. Jønsson . 2022. “Avian Gut Microbiomes Taking Flight.” Trends in Microbiology 30, no. 3: 268–280. 10.1016/j.tim.2021.07.003. 34393028

[ece373682-bib-0007] Bolyen, E. , J. R. Rideout , M. R. Dillon , et al. 2019. “Author Correction: Reproducible, Interactive, Scalable and Extensible Microbiome Data Science Using QIIME 2.” Nature Biotechnology 37, no. 8: 852–857. 10.1038/s41587-019-0209-9. PMC7015180 31341288

[ece373682-bib-0008] Borecka, M. , and M. Karas . 2025. “A Comprehensive Review of the Nutritional and Health‐Promoting Properties of Edible Parts of Selected Cucurbitaceae Plants.” Foods 14, no. 7: 1200. 10.3390/foods14071200. 40238346 PMC11989026

[ece373682-bib-0009] Caporaso, J. G. , C. L. Lauber , W. A. Walters , et al. 2011. “Global Patterns of 16S rRNA Diversity at a Depth of Millions of Sequences Per Sample.” Proceedings of the National Academy of Sciences of the United States of America 108, no. 1: 4516–4522. 10.1073/pnas.1000080107. 20534432 PMC3063599

[ece373682-bib-0010] Chevalier, C. , O. Stojanovic , D. J. Colin , et al. 2015. “Gut Microbiota Orchestrates Energy Homeostasis During Cold.” Cell 163, no. 6: 1360–1374. 10.1016/j.cell.2015.11.001. 26638070

[ece373682-bib-0011] Corbel, H. , A. Legros , C. Haussy , L. Jacquin , A. Lacroix , and J. Gasparini . 2016. “Stress Response Varies With Plumage Colour and Local Habitat in Feral Pigeons.” Journal of Ornithology 157: 825–837. 10.1007/s10336-016-1334-8.

[ece373682-bib-0012] Deagle, B. E. , A. C. Thomas , J. C. McInnes , et al. 2019. “Counting With DNA in Metabarcoding Studies: How Should We Convert Sequence Reads to Dietary Data?” Molecular Ecology 28, no. 2: 391–406. 10.1111/mec.14734. 29858539 PMC6905394

[ece373682-bib-0013] Dietz, M. W. , K. D. Matson , M. A. Versteegh , et al. 2022. “Gut Microbiota of Homing Pigeons Shows Summer–Winter Variation Under Constant Diet Indicating a Substantial Effect of Temperature.” Animal Microbiome 4, no. 1: 64. 10.1186/s42523-022-00216-6. 36514126 PMC9749179

[ece373682-bib-0014] Edgar, R. C. 2010. “Search and Clustering Orders of Magnitude Faster Than BLAST.” Bioinformatics 26, no. 19: 2460–2461. 10.1093/bioinformatics/btq461. 20709691

[ece373682-bib-0015] Faith, D. P. 1992. “Conservation Evaluation and Phylogenetic Diversity.” Biological Conservation 61, no. 1: 1–10.

[ece373682-bib-0016] Faith, D. P. , P. R. Minchin , and L. Belbin . 1987. “Compositional Dissimilarity as a Robust Measure of Ecological Distance.” Vegetatio 69: 57–68. 10.1007/BF00038687.

[ece373682-bib-0017] Gadau, A. , M. S. Crawford , R. Mayek , et al. 2019. “A Comparison of the Nutritional Physiology and Gut Microbiome of Urban and Rural House Sparrows ( *Passer domesticus* ).” Comparative Biochemistry and Physiology Part B, Biochemistry & Molecular Biology 237: 110332. 10.1016/j.cbpb.2019.110332. 31461685

[ece373682-bib-0018] Góngora, E. , K. H. Elliott , and L. Whyte . 2021. “Gut Microbiome Is Affected by Inter‐Sexual and Inter‐Seasonal Variation in Diet for Thick‐Billed Murres ( *Uria lomvia* ).” Scientific Reports 11, no. 1: 1200. 10.1038/s41598-020-80557-x. 33441848 PMC7806582

[ece373682-bib-0019] Grond, K. , B. K. Sandercock , A. Jumpponen , and L. H. Zeglin . 2018. “The Avian Gut Microbiota: Community, Physiology and Function in Wild Birds.” Journal of Avian Biology 49, no. 11: e01788. 10.1111/jav.01788.

[ece373682-bib-0020] Gutiérrez‐Galán, A. , C. A. González , and J. M. D. Mercado . 2017. “Woodpigeon *Columba palumbus* Diet Composition in Mediterranean Southern Spain.” Ardeola 64, no. 1: 17–30.

[ece373682-bib-0021] Huson, D. H. , A. F. Auch , J. Qi , and S. C. Schuster . 2007. “MEGAN Analysis of Metagenomic Data.” Genome Research 17, no. 3: 377–386. 10.1101/gr.5969107. 17255551 PMC1800929

[ece373682-bib-0022] Leray, M. , J. Y. Yang , C. P. Meyer , et al. 2013. “A New Versatile Primer Set Targeting a Short Fragment of the Mitochondrial COI Region for Metabarcoding Metazoan Diversity: Application for Characterizing Coral Reef Fish Gut Contents.” Frontiers in Zoology 10, no. 1: 34. 10.1186/1742-9994-10-34. 23767809 PMC3686579

[ece373682-bib-0023] Lewis, W. B. , F. R. Moore , and S. Wang . 2016. “Characterization of the Gut Microbiota of Migratory Passerines During Stopover Along the Northern Coast of the Gulf of Mexico.” Journal of Avian Biology 47, no. 5: 659–668. 10.1111/jav.00954.

[ece373682-bib-0024] Lewis, W. B. , F. R. Moore , and S. Wang . 2017. “Changes in Gut Microbiota of Migratory Passerines During Stopover After Crossing an Ecological Barrier.” Auk: Ornithological Advances 134, no. 1: 137–145. 10.1642/AUK-16-120.1.

[ece373682-bib-0025] Lim, K. N. , M. C. K. Soh , D. Y. W. Leong , A. H. B. Loo , B. P. Y. H. Lee , and K. B. H. Er . 2023. “Proximity to Anthropogenic Food Sources Determine Roosting and Nesting Prevalence of Feral Pigeons ( *Columba livia* ) in a Tropical City.” Ecological Solutions and Evidence 4, no. 2: e12208. 10.1002/2688-8319.12208.

[ece373682-bib-0026] Liukkonen, M. , J. Muriel , J. Martínez‐Padilla , et al. 2024. “Seasonal and Environmental Factors Contribute to the Variation in the Gut Microbiome: A Large‐Scale Study of a Small Bird.” Journal of Animal Ecology 93, no. 10: 1475–1492. 39041321 10.1111/1365-2656.14153

[ece373682-bib-0027] Loo, W. T. , J. Garcia‐Loor , R. Y. Dudaniec , S. Kleindorfer , and C. M. Cavanaugh . 2019. “Host Phylogeny, Diet, and Habitat Differentiate the Gut Microbiomes of Darwin's Finches on Santa Cruz Island.” Scientific Reports 9, no. 1: 18781. 10.1038/s41598-019-55058-9. 31827126 PMC6906294

[ece373682-bib-0028] Lozupone, C. , and R. Knight . 2005. “UniFrac: A New Phylogenetic Method for Comparing Microbial Communities.” Applied and Environmental Microbiology 71, no. 12: 8228–8235. 10.1128/AEM.71.12.8228-8235.2005. 16332807 PMC1317376

[ece373682-bib-0029] Luniak, M. 2004. “Synurbization: Adaptation of Animal Wildlife to Urban Development.” In Proceedings of the 4th International Urban Wildlife Symposium, edited by W. W. Shaw , L. K. Harris , and L. Vandruff , 50–55. University of Arizona.

[ece373682-bib-0030] Lykke, A. M. , and E. A. Padonou . 2019. “Carbohydrates, Proteins, Fats and Other Essential Components of Food From Native Trees in West Africa.” Heliyon 5: e01744. 31193435 10.1016/j.heliyon.2019.e01744 PMC6531672

[ece373682-bib-0031] Maciusik, B. , M. Lenda , and P. Skórka . 2009. “Corridors, Local Food Resources, and Climatic Conditions Affect the Utilization of the Urban Environment by the Black‐Headed Gull *Larus ridibundus* in Winter.” Ecological Research 25: 263–272. 10.1007/s11284-009-0645-5.

[ece373682-bib-0032] Maraci, Ö. , A. Antonatou‐Papaioannou , S. Jünemann , et al. 2021. “The Gut Microbial Composition Is Species‐Specific and Individual‐Specific in Two Species of Estrildid Finches, the Bengalese Finch and the Zebra Finch.” Frontiers in Microbiology 12: 619141. 33679641 10.3389/fmicb.2021.619141 PMC7933042

[ece373682-bib-0033] Martin, M. 2011. “Cutadapt Removes Adapter Sequences From High‐Throughput Sequencing Reads.” EMBnet.Journal 17, no. 1: 10–12. 10.14806/ej.17.1.200.

[ece373682-bib-0034] Matheen, M. I. A. , M. R. Gillings , and R. Y. Dudaniec . 2022. “Dominant Factors Shaping the Gut Microbiota of Wild Birds.” Emu—Austral Ornithology 122, no. 3–4: 255–268. 10.1080/01584197.2022.2114088.

[ece373682-bib-0038] McArdle, B. H. , and M. J. Anderson . 2001. “Fitting Multivariate Models to Community Data: A Comment on Distance‐Based Redundancy Analysis.” Ecology 82, no. 1: 290–297. 10.1890/0012-­9658(2001)082[0290:FMMTCD]2.0.CO;2.

[ece373682-bib-0039] McDougald, L. R. 2020. “Internal Parasites.” In Diseases of Poultry, edited by D. E. Swayne , 14th ed., 1157–1191. Wiley‐Blackwell.

[ece373682-bib-0040] Minchin, P. R. 1987. “An Evaluation of the Relative Robustness of Techniques for Ecological Ordination.” In Theory and Models in Vegetation Science: Proceedings of Symposium, Uppsala, July 8–13, 1985, edited by I. C. Prentice and E. van der Maarel , 89–107. Springer.

[ece373682-bib-0041] Moorhouse‐Gann, R. J. , J. C. Dunn , N. de Vere , et al. 2018. “New Universal ITS2 Primers for High‐Resolution Herbivory Analyses Using DNA Metabarcoding in Both Tropical and Temperate Zones.” Scientific Reports 8, no. 1: 8542. 10.1038/s41598-018-26648-2. 29867115 PMC5986805

[ece373682-bib-0042] Murton, R. K. , and N. J. Westwood . 1966. “The Foods of the Rock Dove and Feral Pigeon.” Bird Study 13, no. 2: 130–146. 10.1080/00063656609476112.

[ece373682-bib-0043] Obukhova, N. Y. 2007. “Polymorphism and Phene Geography of the Blue Rock Pigeon in Europe.” Russian Journal of Genetics 43: 492–501. 10.1134/S1022795407050050. 17633554

[ece373682-bib-0044] Phillips, J. N. , M. Berlow , and E. P. Derryberry . 2018. “The Effects of Landscape Urbanization on the Gut Microbiome: An Exploration Into the Gut of Urban and Rural White‐Crowned Sparrows.” Frontiers in Ecology and Evolution 6: 148. 10.3389/fevo.2018.00148.

[ece373682-bib-0045] Quast, C. , E. Pruesse , P. Yilmaz , et al. 2013. “The SILVA Ribosomal RNA Gene Database Project: Improved Data Processing and Web‐Based Tools.” Nucleic Acids Research 41, no. D1: D590–D596. 10.1093/nar/gks1219. 23193283 PMC3531112

[ece373682-bib-0046] Ratnasingham, S. , and P. D. N. Hebert . 2007. “BOLD: The Barcode of Life Data System.” Molecular Ecology Notes 7: 355–364. 18784790 10.1111/j.1471-8286.2007.01678.x PMC1890991

[ece373682-bib-0047] Rizwan, A. M. , L. Y. C. Dennis , and C. Liu . 2008. “A Review on the Generation, Determination and Mitigation of Urban Heat Island.” Journal of Environmental Sciences 20, no. 1: 120–128. 10.1016/S1001-0742(08)60019-4. 18572534

[ece373682-bib-0048] Schmiedova, L. , O. Tomasek , H. Pinkasova , T. Albrecht , and J. Kreisinger . 2022. “Variation in Diet Composition and Its Relation to Gut Microbiota in a Passerine Bird.” Scientific Reports 12, no. 1: 3787. 10.1038/s41598-022-07761-4. 35260644 PMC8904835

[ece373682-bib-0049] Schumm, Y. R. , J. F. Masello , J. Vreugdenhil‐Rowlands , D. Fischer , K. Hillerich , and P. Quillfeldt . 2023. “Diet Composition of Wild Columbiform Birds: Next‐Generation Sequencing of Plant and Metazoan DNA in Faecal Samples.” Science of Nature 110, no. 4: 38. 10.1007/s00114-023-01863-8 PMC10363069 37480393

[ece373682-bib-0050] Shor, E. K. , S. P. Brown , and D. A. Freeman . 2020. “A Novel Role for the Pineal Gland: Regulating Seasonal Shifts in the Gut Microbiota of Siberian Hamsters.” Journal of Pineal Research 69, no. 4: e12696. 10.1111/jpi.12696. 32969515

[ece373682-bib-0051] Song, S. J. , J. G. Sanders , F. Delsuc , J. M. Blanton , V. J. McKenzie , and R. Knight . 2020. “Comparative Analyses of Vertebrate Gut Microbiomes Reveal Convergence Between Birds and Bats.” mBiom 11, no. 1: 1–14. 10.1128/mBio.02901-19 PMC6946802 31911491

[ece373682-bib-0052] Tang, K. , L. Tao , Y. Wang , et al. 2023. “Temporal Variations in the Gut Microbiota of the Globally Endangered Sichuan Partridge ( *Arborophila rufipectus* ): Implications for Adaptation to Seasonal Dietary Change and Conservation.” Applied and Environmental Microbiology 89, no. 6: e00747‐23. 10.1128/aem.00747-23. 37272815 PMC10305732

[ece373682-bib-0053] Teyssier, A. , E. Matthysen , N. S. Hudin , L. de Neve , J. White , and L. Lens . 2020. “Diet Contributes to Urban‐Induced Alterations in Gut Microbiota: Experimental Evidence From a Wild Passerine.” Proceedings of the Royal Society B: Biological Sciences 287, no. 1920: 20192182. 10.1098/rspb.2019.2182. PMC7031670 32019440

[ece373682-bib-0054] Uehling, J. J. , and J. L. Houtz . 2025. “Gut Microbiome–Diet Interactions in Wild Birds.” Journal of Avian Biology 2025, no. 5: e03456. 10.1002/jav.03456.

[ece373682-bib-0055] van Veelen, H. P. J. , J. Falcao Salles , K. D. Matson , and B. I. Tieleman . 2020. “Microbial Environment Shapes Immune Function and Cloacal Microbiota Dynamics in Zebra Finches *Taeniopygia guttata* .” Animal Microbiome 2: 17. 10.1186/s42523-020-00043-5. 33499970 PMC7807698

[ece373682-bib-0056] van Veelen, H. P. J. , J. D. Ibañez‐Álamo , N. P. C. Horrocks , M. A. Versteegh , and B. I. Tieleman . 2023. “Cloacal Microbiota Are Biogeographically Structured in Larks From Desert, Tropical and Temperate Areas.” BMC Microbiology 23, no. 1: 40. 10.1186/s12866-023-02778-2. 36765278 PMC9921332

[ece373682-bib-0057] van Veelen, H. P. J. , J. F. Salles , K. D. Matson , G. S. van Doorn , M. van der Velde , and B. I. Tieleman . 2022. “The Microbial Environment Modulates Non‐Genetic Maternal Effects on Egg Immunity.” Animal Microbiome 4, no. 1: 44. 10.1186/s42523-022-00195-8. 35902980 PMC9331593

[ece373682-bib-0058] van Veelen, H. P. J. , J. F. Salles , and B. I. Tieleman . 2018. “Microbiome Assembly of Avian Eggshells and Their Potential as Transgenerational Carriers of Maternal Microbiota.” ISME Journal 12, no. 5: 1375–1388. 10.1038/s41396-018-0067-3. 29445132 PMC5932060

[ece373682-bib-0059] Verkuil, Y. I. , M. Nicolaus , R. Ubels , et al. 2022. “DNA Metabarcoding Quantifies the Relative Biomass of Arthropod Taxa in Songbird Diets: Validation With Camera‐Recorded Diets.” Ecology and Evolution 12, no. 5: e8881. 10.1002/ece3.8881. 35571761 PMC9077022

[ece373682-bib-0060] Videvall, E. , P. Ruiz‐Limón , J. Martínez‐Padilla , I. Moreno‐Indias , D. Canal , and J. Muriel . 2025. “Fine‐Scale Variation in the Gut Microbiome of the European Pied Flycatcher ( *Ficedula hypoleuca* ) in Central Spain.” Ardeola 73, no. 1: 23–42.

[ece373682-bib-0061] Warton, D. I. , and F. K. C. Hui . 2011. “The Arcsine Is Asinine: The Analysis of Proportions in Ecology.” Ecology 92, no. 1: 3–10. 10.1890/10-0340.1. 21560670

[ece373682-bib-0062] Wolff, S. M. , M. J. Ellison , Y. Hao , et al. 2017. “Diet Shifts Provoke Complex and Variable Changes in the Metabolic Networks of the Ruminal Microbiome.” Microbiome 5: 117. 10.1186/s40168-017-0349-1. 28595639 PMC5465553

[ece373682-bib-0063] Xiao, K. , Y. Fan , Z. Zhang , et al. 2021. “Covariation of the Fecal Microbiome With Diet in Nonpasserine Birds.” mSphere 6, no. 3: e00197‐21. 10.1128/mSphere.00197-21. PMC8125056 33980682

[ece373682-bib-0064] Zhu, H. , G. Li , J. Liu , et al. 2022. “Gut Microbiota Is Associated With the Effect of Photoperiod on Seasonal Breeding in Male Brandt's Voles ( *Lasiopodomys brandtii* ).” Microbiome 10, no. 1: 194. 10.1186/s40168-022-01396-9. 36376894 PMC9664686

[ece373682-bib-0065] Zhu, L. , R. Liao , N. Wu , G. Zhu , and C. Yang . 2019. “Heat Stress Mediates Changes in Fecal Microbiome and Functional Pathways of Laying Hens.” Applied Microbiology and Biotechnology 103, no. 1: 461–472. 10.1007/s00253-018-9465-8. 30368579

[ece373682-bib-0066] Zhu, Y. , Y. Li , H. Yang , et al. 2021. “Establishment of Gut Microbiome During Early Life and Its Relationship With Growth in Endangered Crested Ibis ( *Nipponia nippon* ).” Frontiers in Microbiology 12: 723682. 10.3389/fmicb.2021.723682. 34434183 PMC8382091

