# Supplementary material for: Seasonal Dynamics of the Gut Microbiome in Urban Feral Pigeons Are Associated With Environmental Conditions, Not With Diet Shifts
Source: Ecol Evol. 2026 May 13;16(5):e73682. doi: 10.1002/ece3.73682 (PMC13171250; doi:10.1002/ece3.73682)
Supplement: Supplementary file 1 — Table S1: The water content and weight of each material in diet mock samples. Table S2: Outer model specifications for the Partial Least Squares Path Modeling (PLS‐PM) analysis, including latent variables, their corresponding manifest variables, data processing procedures, and the proportion of variance explained. Table S3: Results of factorial ANOVA assessing the main and interactive effects of Season and Location on multiple alpha diversity metrics, including richness, Shannon diversity, and Faith's phylogenetic diversity. Table S4: Permutational multivariate analysis of variance (PERMANOVA) results showing the effects of Season, Location, and their interaction on multiple beta diversity metrics (Jaccard, Bray–Curtis, Unweighted UniFrac, and Weighted UniFrac). Table S5: Generalized linear model (GLM) results testing the effects of season, location, and their interaction on the logit‐transformed relative abundances of the four dominant bacterial phyla. Table S6: Generalized linear model (GLM) results testing the effects of season, location, and their interaction on the logit‐transformed relative abundances of the four dominant bacterial genera. Table S7: Sensitivity of PLS‐PM path coefficients to alternative measurement specifications (bootstrap percentile 95% CI, 5000 resamples). Table S8: Distance‐based redundancy analysis (dbRDA) of gut microbiome composition identifies independent effects of temperature, location, and sex. Table S9: Unique contributions of environmental, diet, host, and location variables were tested using partial redundancy analysis (partial RDA) with permutation tests. Figure S1: Comparison of the expected proportions (EX) of four taxa (Fabaceae, peas; Solanaceae, potato; Taraxacum, dandelion; Triticum, wheat) versus the actual proportions (RE) in mock samples. EX shows the proportions of the dry mass of the two taxa for each mock sample (see Table S1). RE shows the proportions of the actual relative abundances of the taxa obtained in each mo [file ECE3-16-e73682-s001.docx]

**Supporting information**

**Appendix Information:**

Drying procedure:

The water content of the samples (peas, bread, French fries, and dandelion) was determined following a standard oven-drying protocol. All materials were grounded separately and stored in 2 mL centrifuge tubes, from which subsamples were taken for analysis. First, the oven was preheated to 103 °C, and empty weighing cups were dried for 30 minutes, cooled to room temperature in a desiccator, and weighed to obtain the mass of the cup (*Mcup*). The samples were then placed in the cups, evenly spread, and weighed again to determine the initial combined mass (*M1*). The cups with samples were dried in the oven for 4 hours (timing started when the oven reached 103 °C), cooled in a desiccator, and weighed to obtain *M2*. They were then dried for an additional 30 minutes, cooled, and weighed again to record M3. The difference between *M2* and *M3* was checked to ensure it was ≤ 0.1% of the starting mass (i.e., *M1 – Mcup*). The 0.1% tolerance (*M_0.1_*) was calculated as: *M*_0.1_=$\frac{(\left( M2-Mcup \right)-\left( M3-MCup \right))}{(M1-Mcup)} \times100\%$. If the difference met this requirement, the water content (*W*) of the samples was calculated as: $W=\frac{(\left( M1-Mcup \right)-\left( M3-MCup \right))}{(M1-Mcup)} \times100\%$. If not, the drying procedure was repeated in 30-minute intervals until the criterion was satisfied.

PLS-PM:

To elucidate the contributions of the environmental factors, diet, and host traits to gut microbiota diversity, we used Partial Least Squares Path Modeling (PLS-PM) with the plspm package (v0.4.9) (Sanchez, 2013) to build a structural equation model incorporating latent variable relationships for a comprehensive interpretation. We hypothesized that both direct and indirect effects of the environmental factors influence gut microbiota diversity. The indirect effects are proposed to operate through changes in the pigeons’ diet and host traits, ultimately shaping the gut microbial community. To ensure theoretical validity, four latent variables were defined (Table S2): environmental variation (Temperature_PC1 (daily temperature), and Prec_PC1 (daily precipitation)); diet composition (Diet_PCoA1 (diet composition from Cailliez-corrected Jaccard distances), and diet plant species richness); host traits (Body Condition Index (BCI), calculated as residuals of body mass on wing and head bill length, and Sex); and microbiome composition (Microbiome_PCoA1 from the Jaccard PCoA, and ASV richness). Model fit was assessed using the Goodness-of-Fit index and unidimensionality measures, and path significance was tested by bootstrap resampling (5,000 iterations).

Given the effective sample size (n = 59), we specified an a priori, parsimonious structural model and assessed the stability of inferences using sensitivity analyses. Specifically, we compared alternative measurement specifications for latent constructs, including an all-reflective model (Mode A; AAAA), a mixed model treating Environment and Diet as formative composites (Mode B) while retaining Host and Microbiome as reflective (BBAA), and an all-formative model (Mode B; BBBB). Path coefficients and p-values were obtained from the inner model; sensitivity results are reported in Supplementary Table S7.

Sensitivity analyses showed that the inference regarding diet was robust to alternative PLS-PM specifications. The Diet → Microbiome path was non-significant across all three measurement models (Table S7). In contrast, the Environment → Microbiome path remained significant in all specifications (Table S7). While the sign of some paths varied across models due to construct scaling, the presence/absence of effects was consistent, supporting the conclusion that environmental variation showed a stronger association with microbiome composition than diet metrics as measured here.

Reference

Sanchez, G. (2013). *PLS path modeling with R*. Trowchez Editions.

Table S1 The water content and weight of each material in diet mock samples.

| Mock  sample | Food | Water content (%) | Material content (%) | Dry Mass (g) | Wet Mass (g) |
| --- | --- | --- | --- | --- | --- |
| M1 | Peas | 9.22 | 90.78 | 0.18 | 0.20 |
|  | Bread | 13.58 | 86.42 | 0.02 | 0.02 |
| M2 | Peas | 9.22 | 90.78 | 0.10 | 0.11 |
|  | Bread | 13.58 | 86.42 | 0.10 | 0.13 |
| M3 | Peas | 9.22 | 90.78 | 0.02 | 0.02 |
|  | Bread | 13.58 | 86.42 | 0.18 | 0.21 |
| M4 | Peas | 9.22 | 90.78 | 0.18 | 0.20 |
|  | Fries | 40.63 | 59.37 | 0.02 | 0.03 |
| M5 | Peas | 9.22 | 90.78 | 0.10 | 0.11 |
|  | Fries | 40.63 | 59.37 | 0.10 | 0.17 |
| M6 | Peas | 9.22 | 90.78 | 0.02 | 0.02 |
|  | Fries | 40.63 | 59.37 | 0.18 | 0.30 |
| M7 | Peas | 9.22 | 90.78 | 0.18 | 0.20 |
|  | Dandelion | 80.36 | 19.64 | 0.02 | 0.10 |
| M8 | Peas | 9.22 | 90.78 | 0.10 | 0.11 |
|  | Dandelion | 80.36 | 19.64 | 0.10 | 0.51 |
| M9 | Peas | 9,22 | 90.78 | 0.02 | 0.02 |
|  | Dandelion | 80.36 | 19.64 | 0.18 | 0.92 |
| M10 | Bread | 13.58 | 86.42 | 0.18 | 0.21 |
|  | Dandelion | 80.36 | 19.64 | 0.02 | 0.10 |
| M11 | Bread | 13.58 | 86.42 | 0.10 | 0.12 |
|  | Dandelion | 80.36 | 19.64 | 0.10 | 0.51 |
| M12 | Bread | 13.58 | 86.42 | 0.02 | 0.02 |
|  | Dandelion | 80.36 | 19.64 | 0.18 | 0.92 |


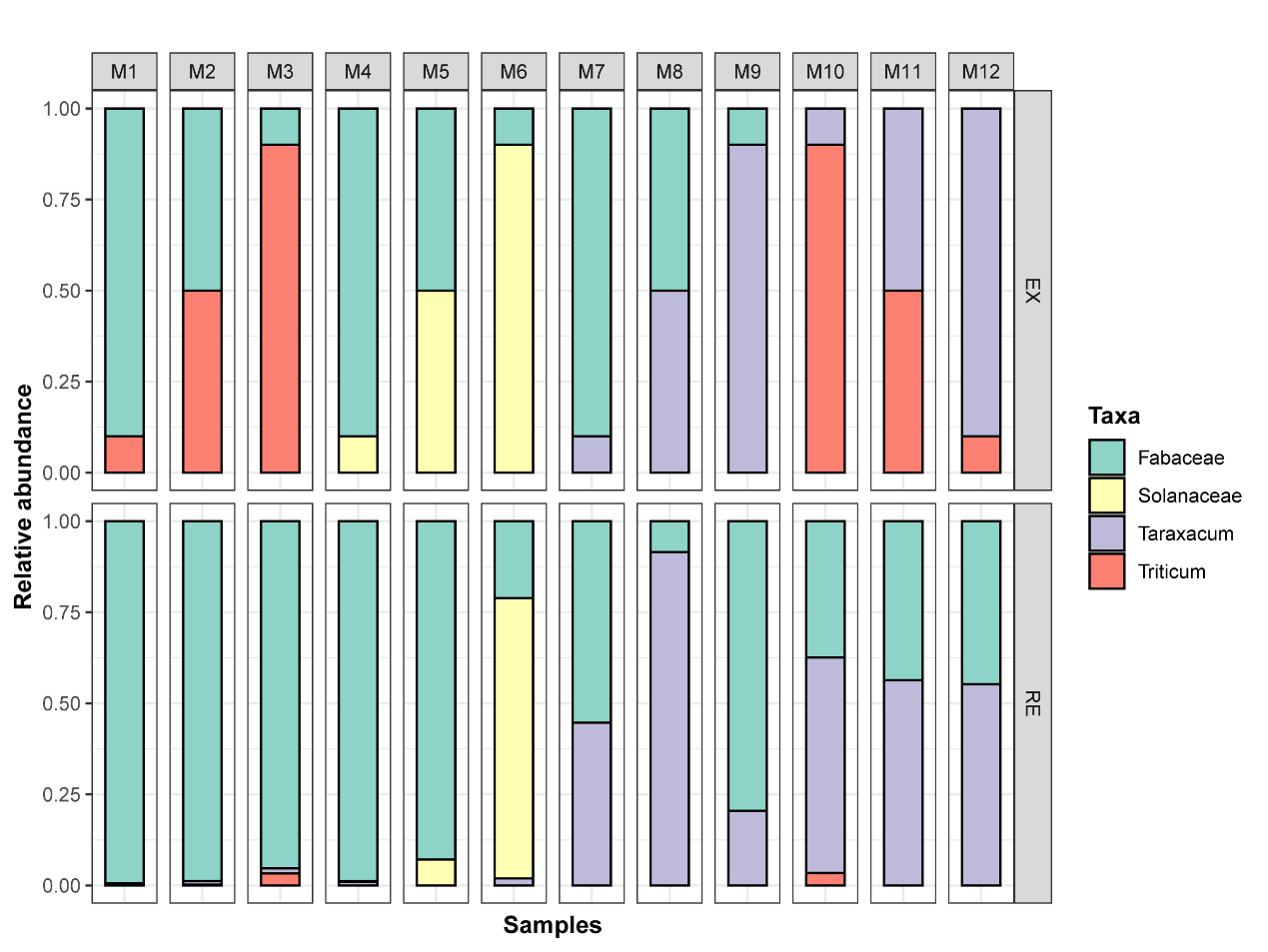


Figure S1 Comparison of the expected proportions (EX) of four taxa (Fabaceae, peas; Solanaceae, potato; Taraxacum, dandelion; Triticum, wheat) versus the actual proportions (RE) in mock samples. EX shows the proportions of the dry mass of the two taxa for each mock sample (see Table S1). RE shows the proportions of the actual relative abundances of the taxa obtained in each mock sample after sequencing. Different colors indicate different taxa.

Table S2 Outer model specifications for the Partial Least Squares Path Modeling (PLS-PM) analysis, including latent variables, their corresponding manifest variables, data processing procedures, and the proportion of variance explained.

| **Latent variable** | **Manifest Variable** | **Data processing** | **Variance explained** |
| --- | --- | --- | --- |
| Environmental variation | Temperature_PC1  Prec_PC1 | Daily mean (TG), minimum (TN), and maximum (TX) temperatures were standardized and subjected to a principal component analysis (PCA). The first principal component (Temperature_PC1) was retained. Precipitation duration (DR) and daily precipitation amount (RH) were also standardized and analyzed using PCA (Prec_PC1). However, since Prec_PC1 showed the lowest loading (-0.09) and weight (-0.05) in the model, it was excluded from the final analysis. | Temperature_PC1 explained 96 % of the variance. Prec_PC1 explained 75 % of the variance. |
| Diet composition | Observed species richnessPCoA1 (Jaccard) | Presence–absence diet data were converted to Jaccard distances and analyzed via PCoA with Cailliez correction to avoid negative eigenvalues. The first axis (PCoA1) was retained. | PCoA1 explained 8.30% of the variance; observed richness represents α-diversity. |
| Host traits | BCI index  Sex | Body condition index (BCI) was calculated as residuals from a linear regression of body mass on wing length and head–bill length. Sex was included as a categorical variable (male: 0, female: 1). | BCI reflects relative body condition; sex accounts for potential sex-related variation. |
| Microbiome composition | Observed species richness  PCoA1 (Weighted UniFrac) | Relative abundance data were used to calculate Weighted UniFrac distances, followed by PCoA. The first axis (PCoA1) was retained. | PCoA1 explained 35.6% of the variance; observed richness represents α-diversity. |

Table S3 Results of factorial ANOVA assessing the main and interactive effects of Season and Location on multiple alpha diversity metrics, including richness, Shannon diversity, and Faith’s phylogenetic diversity.

| Alpha diversity metrics | Factors | df | F | P |
| --- | --- | --- | --- | --- |
| Richness | Season | 57 | 6.69 | **0.01** |
|  | Location | 55 | 1.04 | 0.36 |
|  | Season * Location | 53 | 3.22 | **0.04** |
| Shannon | Season | 57 | 2.27 | 0.13 |
|  | Location | 55 | 1.28 | 0.28 |
|  | Season * Location | 53 | 1.66 | 0.19 |
| Faith’s PD | Season | 57 | 2.80 | 0.10 |
|  | Location | 55 | 2.07 | 0.13 |
|  | Season * Location | 53 | 1.15 | 0.32 |

Table S4 Permutational multivariate analysis of variance (PERMANOVA) results showing the effects of Season, Location, and their interaction on multiple beta diversity metrics (Jaccard, Bray–Curtis, Unweighted UniFrac, and Weighted UniFrac).

| Beta diversity metrics | Factors | R²% | F | P |
| --- | --- | --- | --- | --- |
| Jaccard | Season | 2.59 | 1.55 | **0.02** |
|  | Location | 5.71 | 1.72 | **0.002** |
|  | Season * Location | 0.04 | 1.26 | 0.06 |
| Bray–Curtis | Season | 3.35 | 1.98 | **0.02** |
|  | Location | 7.06 | 2.20 | **0.002** |
|  | Season * Location | 4.54 | 1.41 | 0.06 |
| Unweighted UniFrac | Season | 2.21 | 1.36 | 0.20 |
|  | Location | 6.09 | 1.88 | **0.01** |
|  | Season * Location | 5.73 | 1.77 | 0.06 |
| Weighted UniFrac | Season | 3.69 | 2.32 | 0.51 |
|  | Location | 7.26 | 2.29 | 0.40 |
|  | Season * Location | 4.98 | 1.57 | 0.55 |

Table S5 Generalized linear model (GLM) results testing the effects of season, location, and their interaction on the logit-transformed relative abundances of the four dominant bacterial phyla.

| Phyla | Factors | df | F | P |
| --- | --- | --- | --- | --- |
| Firmicutes | Season | 57 | 3.87 | 0.05 |
|  | Location | 55 | 2.14 | 0.13 |
|  | Season * Location | 53 | 1.54 | 0.22 |
| Actinobacteria | Season | 57 | 5.44 | **0.02** |
|  | Location | 55 | 0.15 | 0.86 |
|  | Season * Location | 53 | 2.68 | 0.08 |
| Proteobacteria | Season | 57 | 0.72 | 0.40 |
|  | Location | 55 | 0.13 | 0.88 |
|  | Season * Location | 53 | 0.50 | 0.61 |
| Tenericutes | Season | 57 | 0.57 | 0.45 |
|  | Location | 55 | 4.96 | **0.01** |
|  | Season * Location | 53 | 0.75 | 0.48 |

Table S6 Generalized linear model (GLM) results testing the effects of season, location, and their interaction on the logit-transformed relative abundances of the four dominant bacterial genera.

| Genera | Factors | df | F | P |
| --- | --- | --- | --- | --- |
| *Lactobacillus* | Season | 57 | 7.47 | **0.01** |
|  | Location | 55 | 3.13 | 0.05 |
|  | Season * Location | 53 | 0.50 | 0.61 |
| *Enterococcus* | Season | 57 | 5.86 | **0.02** |
|  | Location | 55 | 0.64 | 0.53 |
|  | Season * Location | 53 | 3.18 | **0.04** |
| *Corynebacterium* | Season | 57 | 2.00 | 0.16 |
|  | Location | 55 | 5.52 | **0.01** |
|  | Season * Location | 53 | 0.73 | 0.49 |
| *Shigella* | Season | 57 | 1.79 | 0.19 |
|  | Location | 55 | 1.80 | 0.17 |
|  | Season * Location | 53 | 1.03 | 0.36 |

Table S7 Sensitivity of PLS-PM path coefficients to alternative measurement specifications (bootstrap percentile 95% CI, 5000 resamples).

| Path | AAAA β (95% CI) | AAAA p | BBAA β (95% CI) | BBAA p | BBBB β (95% CI) | BBBB p |
| --- | --- | --- | --- | --- | --- | --- |
| Environment → Diet | 0.161 (-0.356, 0.391) | 0.222 | 0.312 (-0.496, 0.507) | **0.016** | 0.296 (-0.492, 0.501) | **0.023** |
| Environment → Host | -0.265 (-0.503, 0.398) | **0.047** | -0.239 (-0.502, 0.428) | 0.085 | -0.249 (-0.502, 0.420) | 0.072 |
| Environment → Microbiome | -0.320 (-0.5, 0.607) | **0.015** | -0.302 (-0.507, 0.634) | **0.026** | 0.454 (-0.521, 0.672) | **0.0006** |
| Diet → Host | -0.043 (-0.35, 0.344) | 0.744 | -0.007 (-0.353, 0.363) | 0.958 | -0.007 (-0.355, 0.366) | 0.957 |
| Diet → Microbiome | -0.127 (-0.415, 0.412) | 0.308 | -0.096 (-0.436, 0.436) | 0.458 | 0.015 (-0.367, 0.340) | 0.900 |
| Host → Microbiome | 0.166 (-0.372, 0.415) | 0.195 | 0.179 (-0.381, 0.413) | 0.161 | -0.150 (-0.368, 0.327) | 0.214 |

Note: AAAA = all constructs reflective (Mode A); BBAA = Environment & Diet formative (Mode B), Host & Microbiome reflective (Mode A); BBBB = all constructs formative (Mode B). Path coefficients are estimates from plspm, with percentile bootstrap 95% confidence intervals based on 5000 resamples.

Table S8 Distance-based redundancy analysis (dbRDA) of gut microbiome composition identifies independent effects of temperature, location, and sex.

|  | Predictors | df | F | P |
| --- | --- | --- | --- | --- |
| Full model | Temp_PC1 | 1 | 1.46 | **0.04** |
|  | Prec_PC1 | 1 | 1.01 | 0.40 |
|  | Location | 2 | 1.55 | **0.003** |
|  | Diet_PC1 | 1 | 0.93 | 0.55 |
|  | Diet_Observed | 1 | 0.90 | 0.68 |
|  | BCI | 1 | 0.77 | 0.91 |
|  | Sex | 1 | 1.41 | **0.045** |
| Partial model (Condition (Location)) | Temp_PC1 | 1 | 1.46 | **0.03** |
|  | Prec_PC1 | 1 | 1.01 | 0.39 |
|  | Diet_PC1 | 1 | 0.93 | 0.58 |
|  | Diet_Observed | 1 | 0.90 | 0.66 |
|  | BCI | 1 | 0.77 | 0.91 |
|  | Sex | 1 | 1.41 | **0.04** |

Note: Marginal permutation tests are shown for a full dbRDA model and a partial dbRDA model conditioning on sampling location. Significant p-values (p < 0.05) are shown in bold.

Table S9 Unique contributions of environmental, diet, host, and location variables were tested using partial redundancy analysis (partial RDA) with permutation tests.

| Fraction | Predictors | Controlled for | F | P |
| --- | --- | --- | --- | --- |
| Environment | Temp_PC1+Prec_PC1 | Diet + Host +Location | 1.57 | **0.01** |
| Diet | Diet_PC1 + Diet_Observed | Environment + Host +Location | 0.85 | 0.76 |
| Host | BCI + Sex | Environment + Diet +Location | 1.38 | 0.054 |
| Location | Location | Environment + Diet + Host | 1.84 | **0.003** |

Note: Unique fractions were tested with partial RDA using 999 permutations, controlling for the other predictor sets as listed. Significant p-values (p < 0.05) are shown in bold.


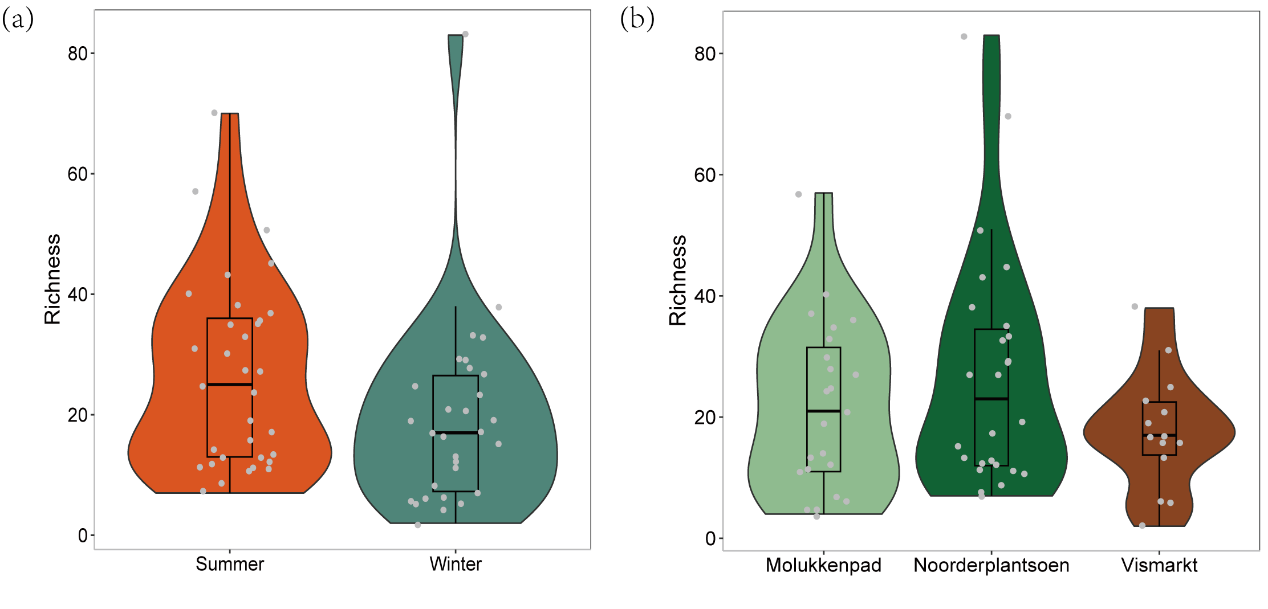


Figure S2 Violin plot showing the overall differences in richness of the diet of feral pigeons between (a) seasons and (b) locations. Dots represent individual samples, while the box show the median, interquartile range, and variability of the data. (summer: n = 33, winter: n = 30; Molukkenpad: n = 23, Noorderplantsoen: n = 26, Vismarkt: n = 14).


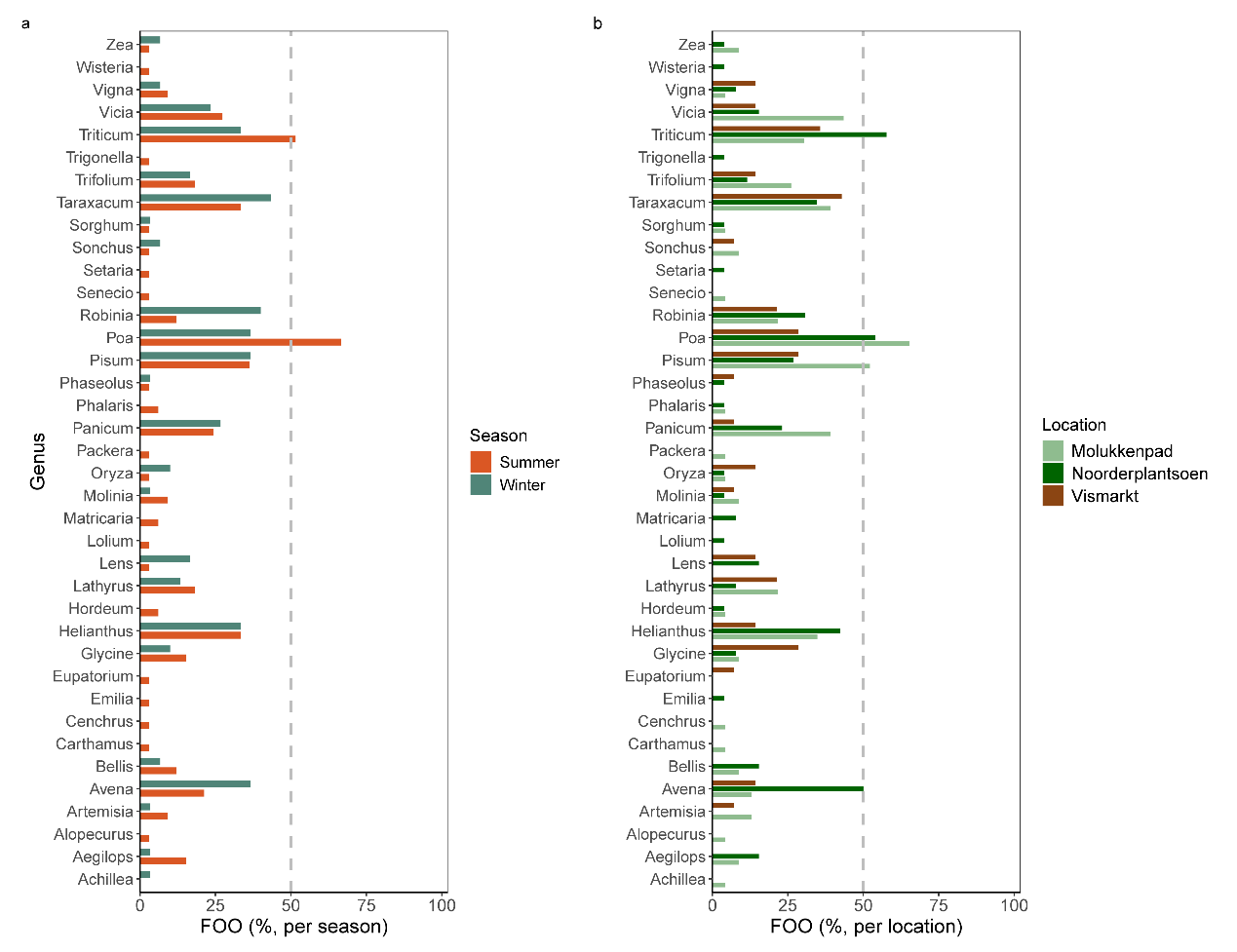


Figure S3 Frequency of occurrence (FOO) of various plant genera consumed by feral pigeons in different (a) seasons and (b) locations. The dashed lines indicate that the genus was found in more than half of the total samples per season (summer: 33, winter: 30) or location (Molukkenpad: 23, Noorderplantsoen: 26, Vismarkt: 14).


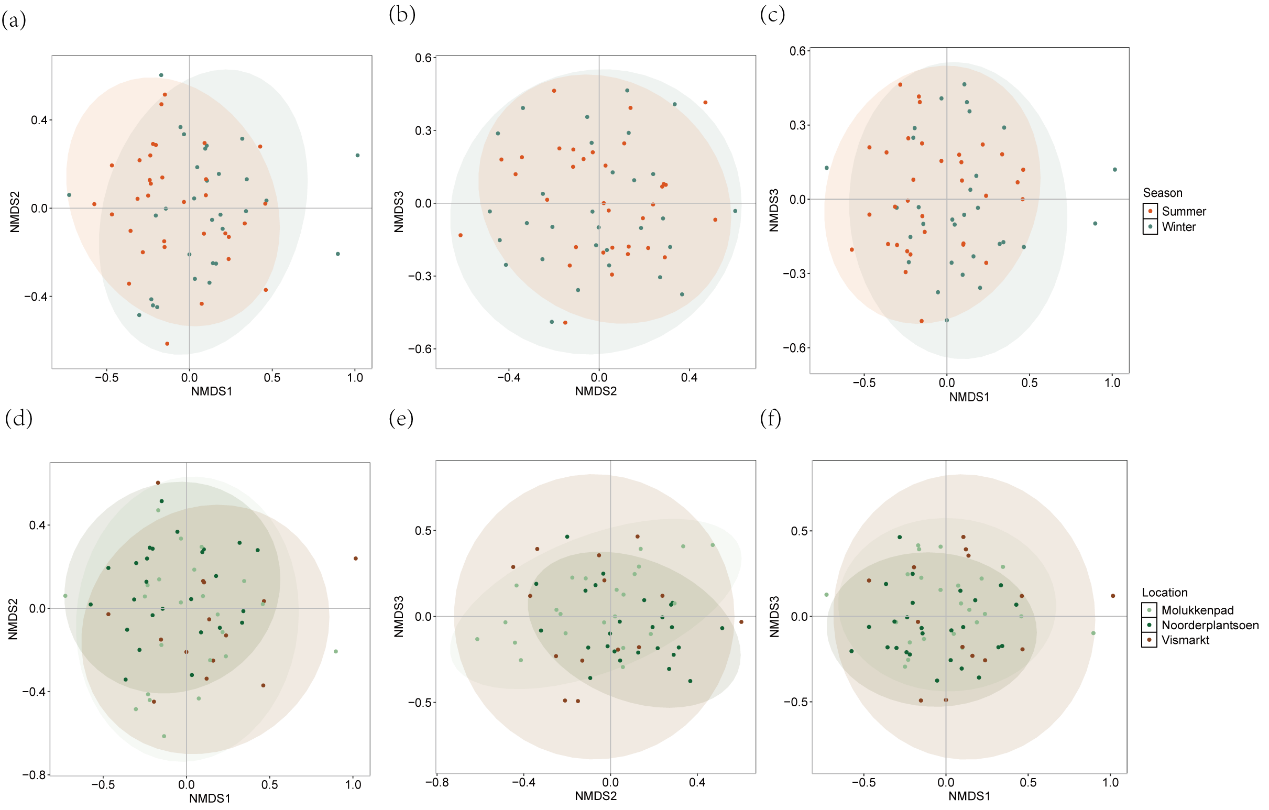


Figure S4 NMDS plots showing patterns of diet composition of feral pigeons across season (a, b, c) and locations (d, e, f) at family level. The ellipses show the 95% confidence regions, indicating the clustering of samples of similar groups. (summer: n = 33, winter: n = 30; Molukkenpad: n = 23, Noorderplantsoen: n = 26, Vismarkt: n = 14).


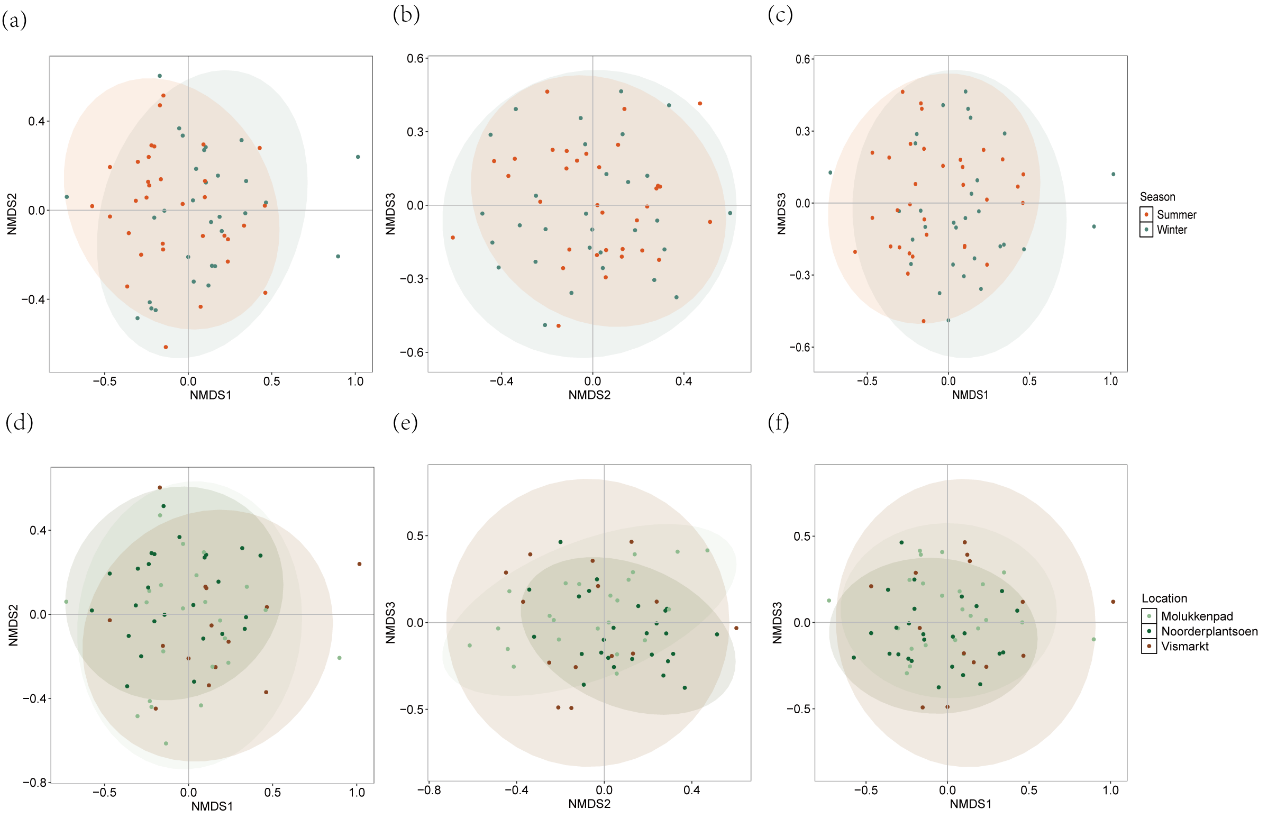


Figure S5 NMDS plots showing patterns of diet composition of feral pigeons across between season (a, b, c) and locations (d, e, f) at genus level. The ellipses show the 95% confidence regions, indicating the clustering of similar samples. (summer: n = 33, winter: n = 30; Molukkenpad: n = 23, Noorderplantsoen: n = 26, Vismarkt: n = 14).
